# Theoretical study of adsorption properties and CO oxidation reaction on surfaces of higher tungsten boride

**DOI:** 10.1038/s41598-024-63676-7

**Published:** 2024-06-04

**Authors:** Aleksandra D. Radina, Viktor S. Baidyshev, Ilya V. Chepkasov, Nikita A. Matsokin, Tariq Altalhi, Boris I. Yakobson, Alexander G. Kvashnin

**Affiliations:** 1 Skolkovo Innovation Center, Skolkovo Institute of Science and Technology, Bolshoy Boulevard 30, bld. 1, Moscow, Russia 121205; 2 Chemistry Department, Taif University, Al Hawiyah, 26571 Taif, Saudi Arabia; 3 Department of Chemistry, Rice University, Houston, TX 77005 USA

**Keywords:** Density functional theory, Surface chemistry

## Abstract

Most modern catalysts are based on precious metals and rear-earth elements, making some of organic synthesis reactions economically insolvent. Density functional theory calculations are used here to describe several differently oriented surfaces of the higher tungsten boride WB_5-x_, together with their catalytic activity for the CO oxidation reaction. Based on our findings, WB_5-x_ appears to be an efficient alternative catalyst for CO oxidation. Calculated surface energies allow the use of the Wulff construction to determine the equilibrium shape of WB_5-x_ particles. It is found that the (010) and (101) facets terminated by boron and tungsten, respectively, are the most exposed surfaces for which the adsorption of different gaseous agents (CO, CO_2_, H_2_, N_2_, O_2_, NO, NO_2_, H_2_O, NH_3_, SO_2_) is evaluated to reveal promising prospects for applications. CO oxidation on B-rich (010) and W-rich (101) surfaces is further investigated by analyzing the charge redistribution during the adsorption of CO and O_2_ molecules. It is found that CO oxidation has relatively low energy barriers. The implications of the present results, the effects of WB_5-x_ on CO oxidation and potential application in the automotive, chemical, and mining industries are discussed.

## Introduction

Heterogeneous catalysis represents a cornerstone of the chemical and energy industries, with ongoing efforts to transition towards more efficient energy use. Catalysts are the key component of heterogeneous catalysis in chemical industries, influencing reaction rates while remaining excluding from the final products. The urgency of catalyst development for important processes such as biomass upgrading, CO_2_ reduction, CO oxidation, and water splitting is driven by the rise in anthropogenic CO_2_ emissions on a global scale^1^.

The most commonly used catalysts for organic synthesis in almost 90% of all chemical industrial reactions are mainly made of noble and rare earth metals^2^, which significantly increases the cost of final products^3^. Reducing the cost of the catalysts while maintaining the same efficiency is a challenging task for science and technology. Researchers worldwide are searching for alternative options that have the same efficiency but a lower cost^4–8^.

One potential alternative is transition metal borides (TMB), which have the potential to serve as catalysts for a wide range of vital chemical processes. The strong boron-boron bonds make these compounds chemically inert^9–11^, with outstanding physical properties^12–15^.

Amongst them, tungsten borides, in particular the higher borides, possess high chemical inertness^16^, corrosion resistance^11^, and good conductivity^17,18^ making them promising electrode materials, components of lithium-ion batteries, and catalysts^18^. Along with the cobalt, molybdenum, nickel, and vanadium borides, tungsten borides were also investigated as potential catalysts for the hydrogen evolution reaction (HER) and CO_2_ reduction^10,16,19^.

Ions of transition metals act as catalytic active sites, while the boron sublattice promotes isolation of active sites and prevents surface passivation. These compounds have a mixture of various types of chemical bonds (ionic, covalent, and metallic) together with the presence of *d-sp*-hybridization between boron and metal atoms leading to a variety of catalytic activities. This gives borides an excellent catalytic properties^16^, although the contribution of the boron sublattice to HER and CO_2_ reduction remains a matter of contention^10^. Therefore, the question of whether transition metal borides can be used heterogeneous catalysis remains open and requires further investigation, which is also highlighted in recent review^20^.

It is also worth noting that TMBs have not previously been studied as catalysts for such an important process as the CO oxidation reaction^21^. The oxidation of harmful CO gas^22^ is the process that takes place in a car engine to improve air quality in cities. Typically, automotive catalysts are made mainly of precious metals such as CeO_2_^23^, Pd^21,24^ and Rh^25^. However, these materials are easily poisoned by the sulfur impurities in petrol^21^, and are difficult to regenerate. Thus, a comprehensive study of the catalytic properties of higher tungsten boride is therefore required to identify all possible catalytic processes in which it could be used, including CO oxidation.

Moreover, it was shown^10,16^ that the catalytic activity of TMBs is dependent on a stoichiometric ratio between boron and metal. In the case of molybdenum borides, the catalytic activity increases as the boron content in the compound increases^19^. The less active compound is Mo_2_B, with isolated boron atoms located in the metallic sublattice. Such dependencies may be explained by the distribution of electron density between the atoms of transition metals and boron. The low adsorption energies on pure transition metal surfaces indicate that catalysts derived from such materials may be susceptible to poisoning. Conversely, the presence of inert boron atoms in TMBs makes the surface more inert, providing chemical and corrosion resistance. Consequently, compounds containing a greater quantity of boron should exhibit enhanced catalytic activity.

In recent years, there has been a great deal of interest in higher metal borides with structure type of MeB_5-x_ (Me = W, Mo), as well as the diborides^15,26–28^. Pak et al.^26^ showed that the powder of higher tungsten boride can be produced efficiently by a vacuumless atmospheric arc plasma method, without the use of high pressure or an inert atmosphere. Such technology could significantly reduce the cost of production of nanosized higher TMBs powders for use in catalysis.

Since the most of metal borides exhibit metallic conductivity^29^, it is possible to consider them as metallic co-catalysts supported by main semiconducting catalyst (i.e. TiO_2_) for different photocatalytic reactions, including the CO oxidation^30–32^, CO_2_ reduction^6,33–35^ and production of hydrogen from various solutions^36–40^. The absence of a band gap allows for the transfer of ‘hot electrons’ between the co-catalyst and the catalyst, resulting in a significant increase in yield for photocatalytic reactions. A comprehensive study of the catalytic activity of transition metals mono- and diborides, and the efficient ways for synthesis of higher tungsten borides, is timely in order to assess the future prospects of these compounds. This is based on the information about the catalytic properties of these compounds.

We present a first-principles investigation of the adsorption properties of WB_5-x_ surfaces against various atmospheric gas agents and their catalytic activity in CO oxidation. A number of differently oriented facets of WB_5_ are considered, and the Wulff construction is used to derive the equilibrium morphology of WB_5_ single crystals. The activity of the exposed surfaces toward CO oxidation is evaluated by DFT calculations. The data obtained allows us to propose the potential application of WB_5-x_ as a catalyst for automotive catalytic converters. This is followed by a discussion of the resistance of its active sites to poisoning by substances involved in the reaction processes^23^.

## Results and discussion

The determination of stable surface orientation and crystal morphology for the considered WB_5_ is presented in detail in the Supporting Information. The results obtained for the B-(010) and W-(101) surfaces are discussed below.

**Figure 1:**
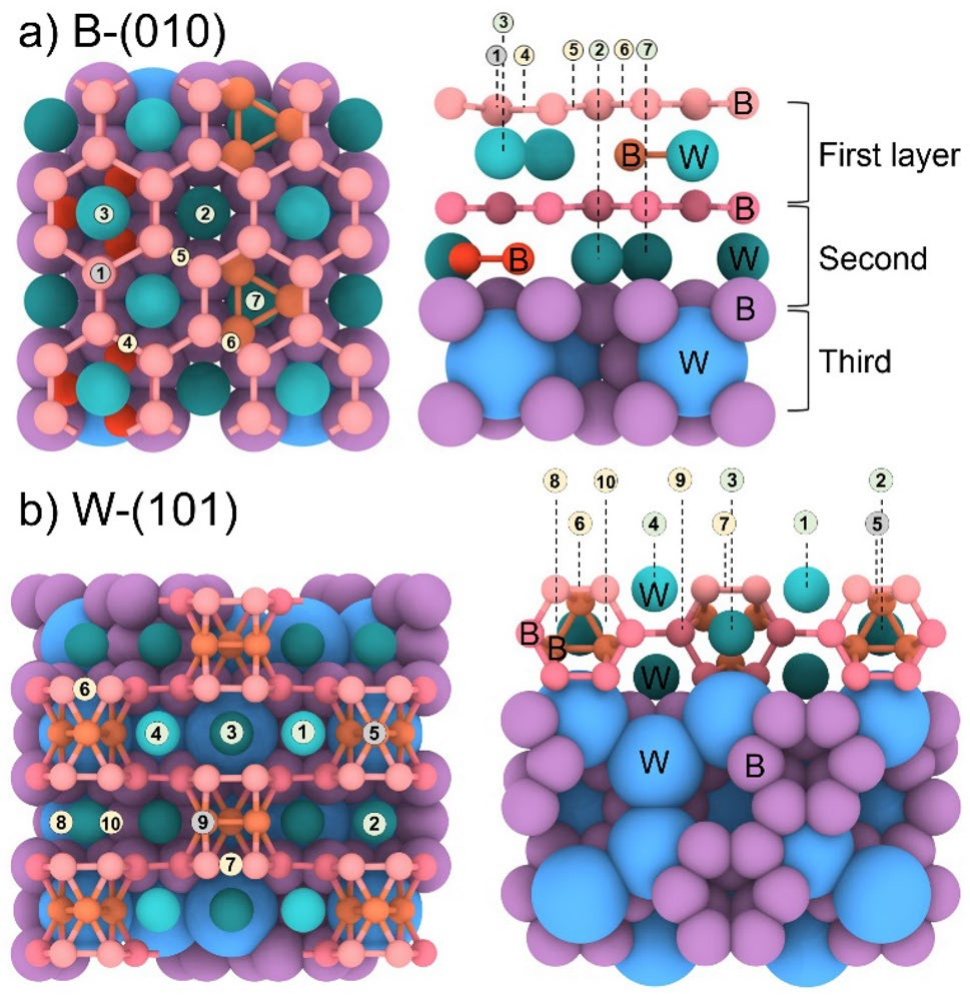
Top and side views of (**a**) B-(010) and (**b**) W-(101) surface with adsorption sites denoted by numbers. Tungsten and boron atoms are differently colored depending on the layer in the structure.

In order to gain a comprehensive understanding of the adsorption of gases on the B-(010) and W-(101) surfaces, it is necessary to consider nonequivalent adsorption sites, as shown in Fig. 1, and Figure S2 in the Supporting Information. In order to elucidate the role of boron in the catalytic process, the adsorption sites (1, 4, 5, and 6 in Fig. 1) over the B atoms or the B-B bonds were examined for the B-(010) surface. Additionally, three additional sites (2, 3, and 7 in Fig. 1) on top of the B-hexagons were considered. The distinguishing feature between these sites is the position of the boron triangle. The site 3 is located on top of the B-hexagon and contains a W atom, with a B-triangle situated underneath. Site 2 is situated above the B-hexagon and contains only a W atom. Site 7 differs from the others, as it is located above the B-hexagon with a B-triangle situated underneath (see Fig. 1). The adsorption sites for W-(101) surface are described in the Supporting Information, Fig. S2.

It should be noted that the crystal structure of higher tungsten boride WB_5-x_ is disordered with respect to the location of B-triangles^26,28^. This leads to compositional variation (0 < x < 2) of WB_5-x_. One of limiting cases is WB_3_ structure with alternation of B and W layers^9,13^ (no B-triangles). Another limiting case is WB_5_, as detailed earlier^13^. The adsorption sites considered in this study include those typical for pure WB_3_ and WB_5_, as well as those for the intermediate compositions, including the sites not typical for the limiting cases.

**Figure 2:**
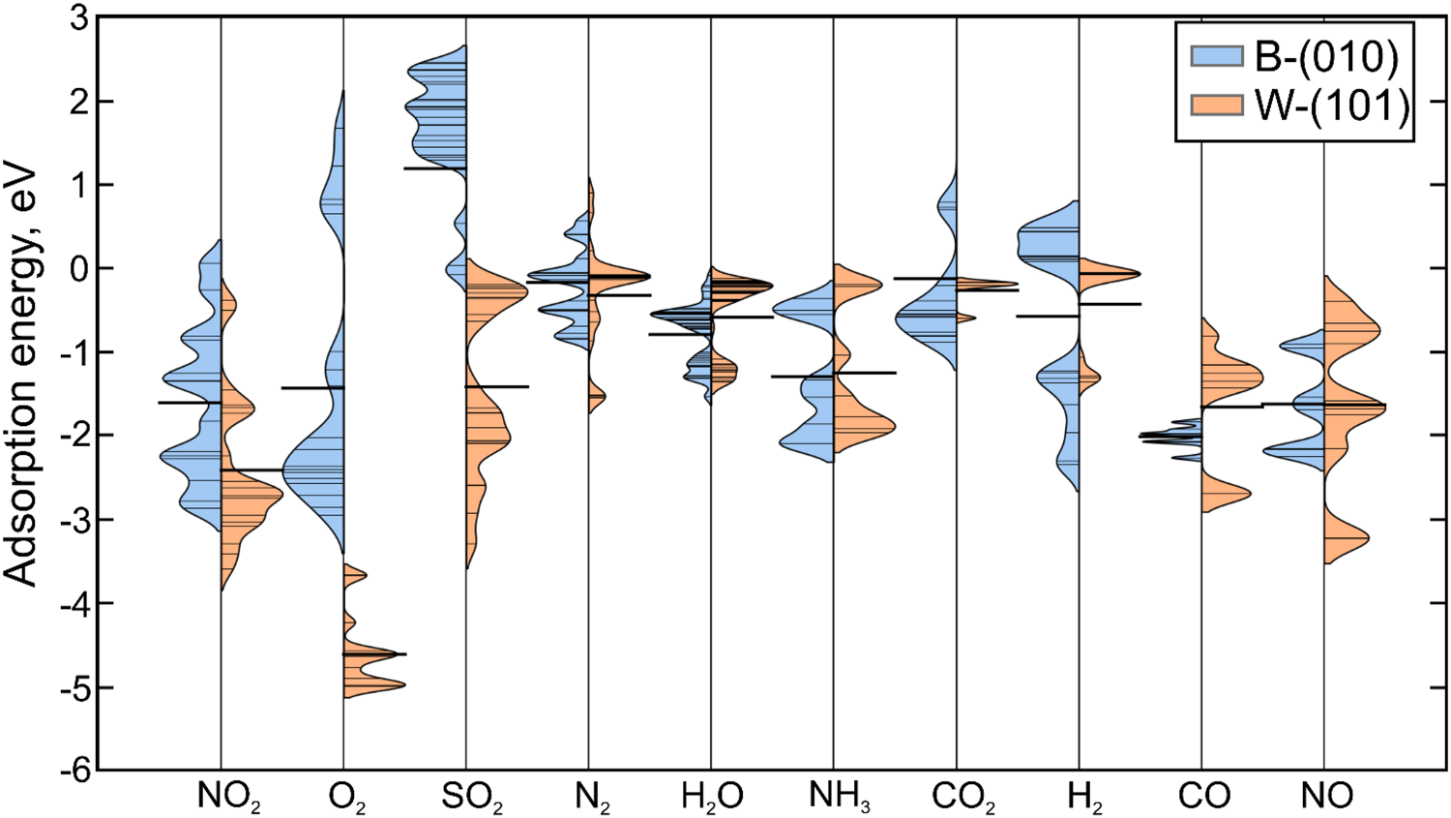
Violine plots showing the distributions of the adsorption energies of considered molecules calculated for all adsorption sites and geometry configurations of adsorbates on B-(010) and W-(101) surfaces. The distributions were obtained by applying 0.35 eV smearing to the calculated adsorption energies. The solid horizontal lines reflect average adsorption energies calculated for each distribution.

In order to gain further insight into the adsorption of gases on the higher tungsten borides, it is necessary to consider all possible configurations of adsorbates on the surfaces. For example, a CO_2_ molecule can adsorb in both linear^41^ and triangular^42,43^ configurations. At the same time, water molecule (H_2_O), with its strong dipole moment, can bind in four different configurations with respect to the surface^44–46^. Thus, the adsorption of ten molecules is considered here: CO, CO_2_, H_2_, N_2_, O_2_, NO, NO_2_, H_2_O, NH_3_, and SO_2_. The total number of considered configurations on B-(010) is as follows: 7 for CO, NO, and NH_3_; 18 for H_2_; 21 for O_2_ and N_2_; 28 for NO_2_, SO_2_ and CO_2_; and 62 for H_2_O molecules. The distributions of adsorption energies for the W-(101) and B-(010) surfaces are shown in Fig. 2 with orange and blue colors, respectively. The total number of considered configurations on the W-(101) surface is: 10 for CO, NO, and NH_3_; 20 for H_2_; 15 for O_2_ and CO_2_; 28 for N_2_, NO_2_, and SO_2_; and 62 for H_2_O molecules.

The adsorption on the B-(010) surface was found to be most favorable at the B-B bonds (sites 4, 5, 6 in Fig. 1) and on top of the B atom (site 1 in Fig. 1). The adsorption energies of all molecules at all considered sites are given in Tables S2 and S3 in the Supporting Information. Possible adsorption configurations of each molecule are given on Figure S5 in the Supporting Information. In contrast to previous study^10^, we find that the surface B atoms are actively involved in the adsorption process. This is corroborated by the relaxation of a molecule originally placed in the center of the B-hexagon, which shifts to the B atom or to the B-B bond, where it remains fixed. Furthermore, during the dissociation of the molecules, the products also bind to the B-B bonds.

**NO** The adsorption of NO molecules occurs vertically, with the N atom situated closer to the surface. The average adsorption energies for the B-(010) and W-(101) surfaces are −1.66 and −1.62 eV, respectively (Fig. 2). The reference data for Cu_20_ clusters^47^ indicates that the adsorption energies are higher, at approximately −0.52 eV. In the case of PbAu, the adsorption energies are 0.72 eV^48^.

**NO**_**2**_ molecules can be adsorbed in two different orientations: parallel to the surface or with the N atom directed towards the surface. If the molecule adsorbs in a parallel orientation and forms a bond with the surface through two atoms, dissociation could occur, particularly in the case of the B-(010) surface. However, none of the dissociated molecules were included in the distributions shown in Fig. 2. Consequently, the average adsorption energies for the B-(010) and W-(101) surfaces are −1.60 and −2.51 eV, respectively. The adsorption energy on the B-(010) surface is comparable to that observed for graphene, with an average value of 1.25 eV^49^.

**NH**_**3**_ molecule predominantly adsorbs on the boron atoms, with average adsorption energies of −1.29 and −1.25 eV for the B-(010) and W-(101) surfaces, respectively (Fig. 2). The obtained numbers are comparable to those found on single-atom-embedded ternary B_3_C_2_P_3_ monolayers (−1.37 eV)^50^. The adsorption energies of NH_3_ on Pt(111) and on Co(0001) are −0.95 and −0.69 eV, respectively, which are higher^51^ but still comparable to that on WB_5-x_.

**N**_**2**_ molecule exhibits a strong affinity for the top of the tungsten atom. The adsorption energies of −0.14 and −0.24 eV for the B-(010) and W-(101) surfaces, respectively, as depicted in Fig. 2, indicate a weak interaction between the molecule and the surface. Consequently, dissociation of this molecule does not occur. The obtained data are comparable to those for Si-doped graphene (−0.15 eV) as reported in ref.^52^. However, the obtained energies are higher than those for other transition metals, such as Mo-doped Fe_2_P (−0.96 eV)^53^.

**H**_**2**_ molecules also primarily interact with tungsten atoms, with average adsorption energies of about −0.57 and −0.54 eV for the B-(010) and W-(101) surfaces, Fig. 2. The number for the W-(101) surface is comparable to that for the Ti_2_AC (A = Al, Ti, Cr, Mn, Fe, Co, Ni, Cu, and Zn)^54^ where it is equal to −0.40 eV. Dissociation of the molecule can only occur at sites on the B-B bond. The energy of the dissociated molecule is represented in Fig. 2 as the energy per atom of the dissociated molecule.

**SO**_**2**_ molecules can be adsorbed in a parallel manner, with the S atom closer to the surface. The interaction between the SO_2_ molecule and the W-(101) surface is stronger than that with the B-(010) surface, as evidenced by the difference in the average adsorption energies of + 1.19 and −1.39 eV, respectively, as shown in Fig. 2. Although dissociation of the molecule during adsorption in a parallel manner could occur while interacting with the tungsten atoms on the W-(101) surface, these cases are not considered in the distributions shown in Fig. 2. The adsorption energy of the SO_2_ molecule is also found to be positive (approximately +0.29 eV) for the TiO_2_ surface^55^, but there are some catalysts, for example metal organic frameworks, where SO_2_ could be adsorbed and the adsorption energy is about −0.59 eV^56^.

**H**_**2**_**O** molecule exhibits four different adsorption configurations relative to the surface. Tables S2 and S3 in the Supporting Information contain all of them. The molecule primarily interacts with boron atoms, which is the reason for its good adsorption on the B-(010) surface with an average adsorption energy of about −0.88 eV. Conversely, it mostly does not adsorb onto the W-(101) surface, with an average adsorption energy of about −0.63 eV. These values of adsorption energies correlate well with those for Cu (−0.33 eV)^57^ and for the Ru (0001) surface, where the adsorption energy is −0.29 eV^45^.

**CO** gas molecules adsorb vertically with the C atom positioned closer to the surface, primarily on the B-B bonds. The average adsorption energies for the B-(010) and W-(101) surfaces are −1.55 and −1.83 eV, respectively, see Fig. 2. These values are considerably lower than those observed for Si-doped graphene (−0.19 eV^52^), suggesting that WB_5-x_ may be a more promising catalyst for reactions initiating by CO gas.

**O**_**2**_ molecule dissociate without any energy barrier in the majority of cases. The energies of the dissociated oxygen atoms are shown in the distribution in Fig. 2, measured in electronvolts per atom. The average adsorption energies are equal to −1.56 and −4.60 eV for the B-(010) and W-(101) surfaces, respectively. The adsorption energy of O_2_ on the W-(101) surface is comparable to that of silicene (−5.38 eV)^58^, where it is also dissociated.

**CO**_**2**_ molecule was analyzed in its linear form, as it exists in the atmosphere. The average energies of its adsorption are approximately −0.37 and −0.7 eV for the B-(010) and W-(101). Figure 2. The average value obtained for the B-(010) surface is comparable to that obtained for the C_6_N_6_ monolayer embedded with Fe single-atom (−0.46 eV)^59^. However, it is not directly comparable with the value obtained for the Si-doped graphene (−0.18 eV)^52^. It is also noteworthy, that the distributions shown in Fig. 2 do not take into account dissociation of the molecule.

The adsorption energies indicate that the CO and NO oxidation reactions are possible on the B-(010) surface, according to both the Eley–Rideal and Langmuir–Hinshelwood mechanisms. The adsorption of the reaction products (NO_2_ and CO_2_) is significantly weaker than the adsorption of the reagents (NO and CO), which suggests the catalytic effectiveness of the B-(010) surface of WB_5_. Furthermore, based on the strong adsorption of NO and CO, as well as the weak adsorption of N_2_ and CO_2_, it can be proposed that WB_5_ could be used as active material for an automotive catalytic converter. In addition to its efficient production^26^, the advantage of WB_5-x_ over currently used catalysts is its resistance to poisoning by sulfur gases, particularly SO_2_^21^, whose adsorption energy is calculated to be positive on this surface, see Fig. 2.

It is important to note that there is a high probability of dissociation of CO_2_ during the adsorption process on both surfaces. This suggests that WB_5-x_ could be a promising catalyst or co-catalyst for the conversion of carbon dioxide into fuels and other vital products^6,33^.

In contrast to the B-(010) surface, the W-(101) surface does not typically bind with water molecules. However, when the water molecule does attach to the surface, the distance between the molecule and the surface is approximately 2.25 Å, resulting in a weak interaction and low adsorption energy. It can therefore be assumed that WB_5-x_ has potential applications as a functional component of filters for the purification of water from heavy metals and halogenated organics, including pesticides^60^. This is because the interaction of water with the catalyst made of WB_5-x_ is not significant.

The stability and adsorption properties of WB_5_ surfaces were examined in the context of the CO reduction reaction on B-(010) and W-(101). The most energetically favorable positions of CO and O_2_ molecules on the surfaces were previously determined.

**Figure 3:**
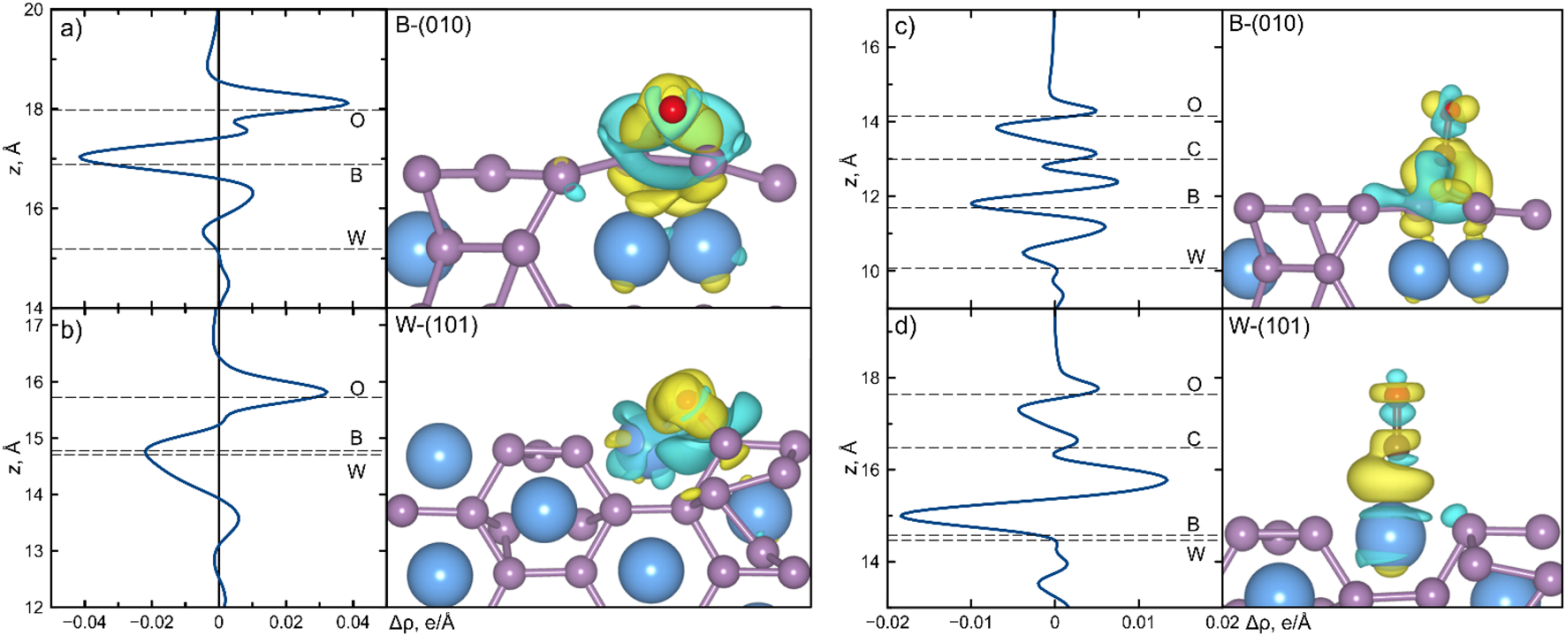
Integrated charge distribution along z-axis for considered (**a**) B-(010) and (**b**) W-(101) surfaces with adsorbed O_2_ molecule, and (**c**, **d**) with adsorbed CO molecule. Spatial charge redistributions on the right panels are plotted with isosurface value of 0.03 e/Å^3^.

In order to gain a deeper understanding of the low adsorption energies of CO and O_2_ molecules the Bader charges^61^ of considered structures were calculated, as illustrated in Fig. 3. It is well-established that there is a charge transfer from the W to B atoms in the tungsten boride due to the differing electronegativity of the two elements. The Bader charge analysis indicates that each W atom of the B-(010) surface loses approximately −1.12 *e*, while on the W-(101) surface each W atom loses −1.05 *e*. It was found that an excess of electrons at each boron atom of the B-(010) surface was equal to + 0.17 e, while a larger excess of + 0.21 *e* was found for the W-(101) surface. Consequently, the differences in charge redistribution on various surfaces are minimal. The direction and magnitude of the charge transfer can be attributed to the smaller work function of tungsten compared to boron^62^. A similar effect was observed for other bicomponent systems with different work functions of constituent elements^63–67^.

The low adsorption energy of CO on the W-(101) surface can be attributed to the CO preference for attaching to positively charged sites, rather than negatively charged ones. This is due to the more pronounced electron donation nature of CO as evidenced by the findings of studies from refs.^68,69^. The positively charged W sites are more favorable for CO adsorption than the negatively charged B sites. In the case of the B-(010) surface the CO donation is 0.13 e, which is much smaller than for the W-(101) surface where it is about 0.54 eV.

The adsorption of the O_2_ molecule was investigated by calculating Bader charge redistributions for oxygen after dissociation on both surfaces (B-(010) and W-(101)). The results demonstrated that the surface donated 1.52 *e* to oxygen in the case of B-(010) and 1.21 *e* in the case of W-(101). It is well known that the O_2_ molecule acts as an electron acceptor and interacts strongly with those sites capable of donating electrons easily to the antibonding orbitals of the O_2_^70^. In our case the sites with B atoms can donate electrons. The electron density difference Δρ (the density of the combined system minus the sum of the densities of the isolated slab and adsorbate at the same positions as in the combined system) for the oxygen molecule is shown in Fig. 3a,b. It is evident that there is a greater redistribution of charge on the surface on B-(010) and that the bond between the surface and oxygen is stronger, as the boron atoms are negatively charged. With regards to the CO molecule on the W-(101) surface, as illustrated in Fig. 3d, the redistribution of charge occurs to a greater extent on the bond between CO and the surface than on the B-(010) surface (Fig. 3c). This indicates that the CO molecule is more strongly bound to the W-(101) surface.

The data indicates that the W-(101) surface is more suitable for the CO oxidation reaction due to the presence of positively charged sites with low CO adsorption energy and negatively charged sites with B atoms, which are conductive to adsorption and dissociation of oxygen.

The number of possible CO oxidation mechanisms is increasing, with a growing number of complex and less probable pathways. There are challenging to identify and explore without the use of detailed computational simulations^21,71^. However, there are two traditional mechanisms that can occur on the surfaces without oxygen atoms.

The first is the Eley–Rideal (ER) mechanism^72,73^. This occurs when the oxygen has a strong adsorption on the surface. This results in the formation of an association between the CO gas molecule and the pre-adsorbed O_2_ molecule.

In contrast, the Langmuir–Hinshelwood (LH) mechanism^24,72^ can proceed when both CO and O_2_ have a strong adsorption on the surface and can move across the surface with a relatively small energy barrier. In this case, the CO molecule moves to the adsorbed O_2_ molecule to form the OCOO complex. This complex then dissociates into the CO_2_ gas molecule and adsorbed O atom, which could react with another CO molecule.

Consequently, the mechanism of CO oxidation is primarily defined by the adsorption possibilities of CO and O_2_. As previously stated, both CO and O_2_ can be readily adsorbed on either the B-(010) or W-(101) surfaces, see Figures S3, S4 in the Supporting Information. Consequently, the CO oxidation reaction can occur through both the ER and LH mechanisms. The O_2_ has a stronger adsorption on both surfaces, thus the ER mechanism is more probable.

The O–O bond in the O_2_ molecule is known to be particularly strong, making it challenging to successfully apply in oxidative chemistry. Different catalysts were considered to overcome the high barrier of O_2_ dissociation, for instance, the Au_x_Pd_x_ clusters^70^. In our case, O_2_ dissociates on both the B-(010) and W-(101) surfaces without any energy barrier, as shown in Figure S3, S4 in the Supporting Information. Consequently, WB_5-x_ could be proposed as the catalyst for environmentally and industrially important reactions such as ethylene epoxidation, hydrocarbon oxidation, and so on^74–76^.

**Figure 4:**
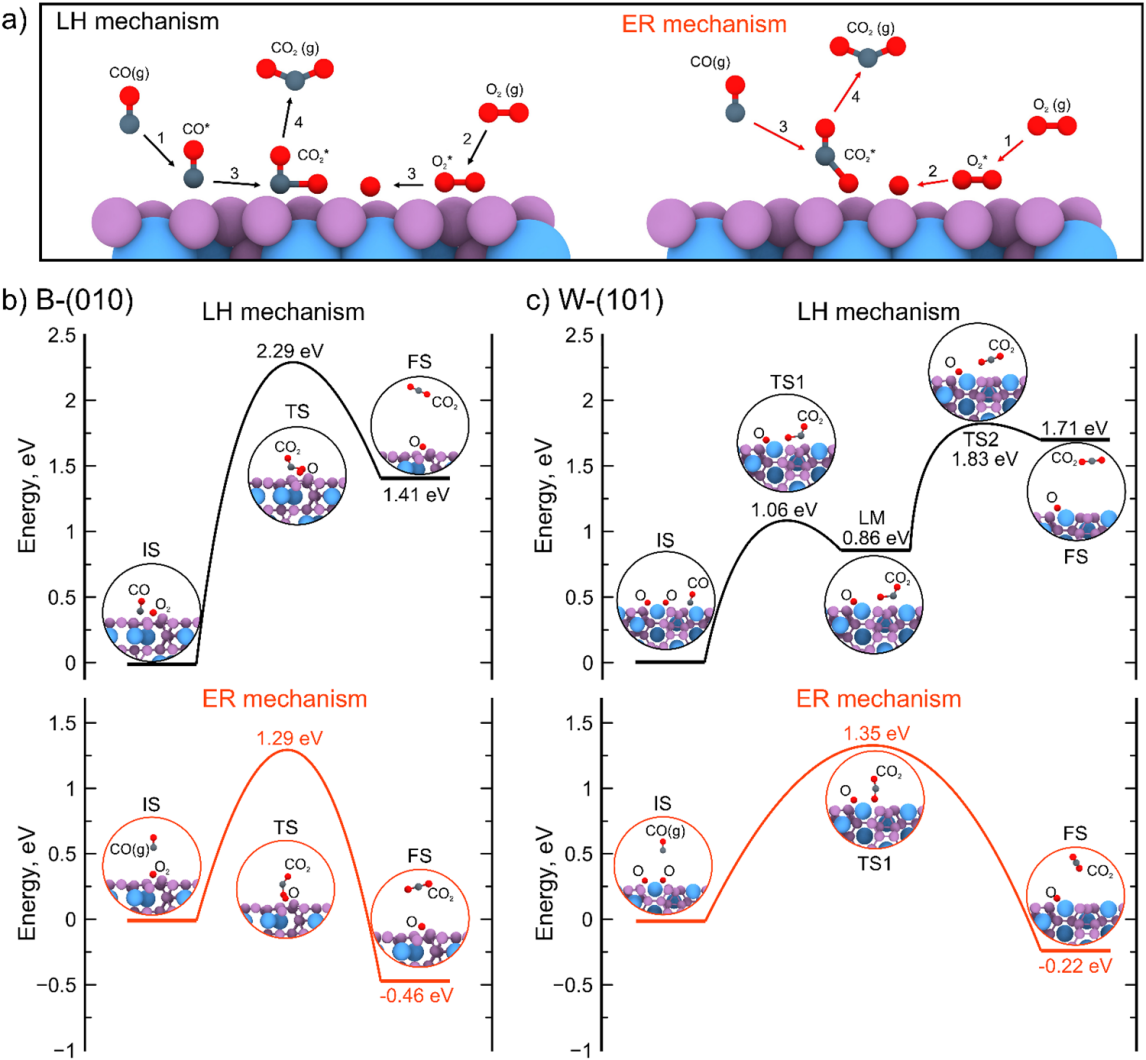
(**a**) Principal scheme of CO oxidation reaction according to Langmuir–Hinshelwood and Eley–Rideal mechanisms. For clarity the only surface of B-(010) is shown. Energy barriers calculated for CO oxidation according to Langmuir–Hinshelwood (black) and Eley–Rideal (red) mechanisms on (**a**) B-(010) and (**b**) W-(101) surfaces.

Thus, in the case of CO oxidation over the WB_5-x_ surfaces, the mechanisms become less complicated and the only difference between the ER and LH mechanisms is the state of CO (gas or pre-adsorbed). We have conducted a detailed study of CO oxidation mechanisms for each surface, as shown in Fig. 4. In the Fig. 4 the schematic illustration of two processes is shown. For LH reaction the energy barriers are shown in the Fig. 4b,c for steps 3,4, while energy barriers for steps 1,2 presented in the Supporting Information (Figures S3, S4).

For B-(010) surface, the rate-limiting step is the bonding of the CO and O*; see transition state (TS) in Fig. 4b. The CO oxidation occurs through the ER and LH mechanisms, with reaction barriers of 1.29 and 2.29 eV, respectively.

In the case of the W-(101) surface, the rate-limiting steps for the different reaction mechanisms are different, as illustrated in Fig. 4c. In the case of CO oxidation via the ER mechanism (red color in Fig. 4c), the rate-limiting step is the bonding between the CO and O* with a reaction barrier of 1.35 eV. Conversely, in the case of CO oxidation via the LH mechanism (black color in Fig. 4c), the rate-limiting step is the CO_2_ release, with an energy barrier of 1.83 eV.

The information obtained regarding the reaction barriers of CO oxidation on the considered surfaces indicates that this reaction will occur on the WB_5-x_ exterior. The major mechanisms of reactions in this case could be both the ER and LH mechanisms. Surprisingly, the W-(101) surface has the lowest energy barrier for CO oxidation of 1.35 eV. Nevertheless, the B-(010) surface, devoid of any metal atoms on the surface, has the lowest energy barrier 1.29 eV for CO oxidation. This minimal difference in energy barriers may indicate the active sites without any metal atoms and the boron participation in the catalytic process.

It is important to note that the energy barrier for the CO oxidation reaction may appear to be higher than that of Au nanoparticles, which is approximately 0.4 eV^77^. However, when considering the overall structure of the noble metal, the range of values is significantly different. Both bulk Au and Pd have energy barriers of approximately 0.93 eV^78,79^ and 1.02 eV^80^, respectively. It should be noted that the O_2_ molecule is adsorbed onto these surfaces in its initial state, and there is an additional energy barrier to dissociate it. The Pt surface exhibits a lower barrier of 0.47–0.63 eV, but it is easily poisoned by oxygen, resulting in an increased reaction barrier of 1.14 eV^81^.

The data presented thus far allows us to conclude that further investigation of transition metal borides as potential catalysts is a promising avenue for future research. Potential modifications to this material include doping with other transition metals, considering nanoparticles, forming single atom alloys, and catalysis on the surfaces.

## Conclusions

In conclusion, a comprehensive investigation of the structural, adsorption, and catalytic properties of surfaces of higher tungsten boride WB_5-x_ was performed with density functional theory. This was done to examine various crystallographic orientations of WB_5_ surfaces. Atomistic structural models allow one to calculate surface energies, as well as adsorption energies and activation barriers of relevant elementary reactions. Two surfaces, B-(010) and W-(101), have been identified as the main determinants of the equilibrium morphology of higher tungsten boride. They exhibit the lowest surface energies and possess exceptional properties with respect to adsorption of gas agents, including CO, CO_2_, H_2_, N_2_, O_2_, NO, NO_2_, H_2_O, NH_3_, SO_2_. The results of the computational adsorption parameters for CO, CO_2_, H_2_, N_2_, O_2_, NO, NO_2_, H_2_O, NH_3_, SO_2_ gas agents on both surfaces indicate that WB_5-x_ has the potential to be used in car catalytic converters and reductors due to its low susceptibility to sulfur gas poisoning. The investigation of the CO oxidation reaction on both surfaces revealed that it may proceed via both the Eley–Rideal and the Langmuir–Hinshelwood mechanisms. This is due to the fact that both CO and O_2_ molecules are easily adsorbed on both of the considered surfaces. The calculated low energy barriers may suggest the involvement of boron active sites in the catalytic process for CO oxidation. The computational study of the catalytic activity of WB_5-x_ surfaces has demonstrated the potential of higher tungsten boride in a variety of applications, including as a catalyst or co-catalyst in filters for the cleaning of industrial exhaust gases and others. This has opened new avenues for research and applications of this unique material.

The primary result of this study is the potential use of borides to enhance the catalytic properties of a group of new catalysts. Although the calculated barrier for CO oxidation is high, a number of options for further development can be proposed, including doping with other metals, consideration of single atom alloy catalysis (SAAC) formation on the surface, cocatalysts, and nanoparticle-supported catalysts. These directions should be further developed, and this study represents a first step in this process.

## Computational details

All the calculations were performed using density functional theory (DFT) as implemented in VASP package^82–84^. The exchange–correlation effects were treated using the generalized gradient approximation (GGA) with the Perdew-Burke-Ernzerhof (PBE) parametrization^85^. The projector augmented-wave (PAW) method^86^ was used to describe the interactions between the ionic core and valence electrons. The cutoff energy of plane waves was set to 500 eV. The partition of the first Brillouin zone into a grid of k-points was carried out within the Monkhorst–Pack scheme^87^ with a resolution of 2π × 0.05 Å^−1^.

We used the crystal structure of WB_5_ model proposed in^13,88^ to construct slabs with surfaces of different crystallographic orientations. The calculated lattice constants for bulk WB_5_ (*Pmmn* space group) are *a* = 5.209 Å, *b* = 6.349 Å, *c* = 8.893 Å, which are consistent with experimental measurements^26,28,89^.

To calculate the surface energy, symmetric slabs of WB_5_ with (001), (010), (100), (110), (101), (111), (130), and (201) crystallographic orientations of the surfaces were created from the relaxed bulk structure, using the atomic simulation environment (ASE)^90^. The slab thickness ranges from 10 to 15 Å depending on orientation. A vacuum region of 25 Å perpendicular to each surface was used to avoid the artificial interactions between periodic images of the slabs.

For the (001), (010), (100), (110), and (101) surfaces two terminations were considered to identify the most stable one. First type was the tungsten termination (W-rich, denoted as W-(*hkl*) surface), and the second type with predominance of boron (B-rich, B-(*hkl*) surface). Thus, 13 different surfaces were constructed to be studied. Each surface energy was calculated as:

1 $$\gamma =\frac{1}{2S}\left[{G}^{slab}\left({W}_{m+n}{B}_{5m+k}\right)-m{G}^{bulk}\left({WB}_{5}\right)-n\mu \left(W\right)-k\mu (B)\right],$$

where $\gamma$ is the surface energy per surface area, ${G}^{slab}\left({W}_{m+n}{B}_{5m+k}\right)$ is the Gibbs free energy of the surface per cell, *m* is quantity of cells in considered slab, ${G}^{bulk}\left({WB}_{5}\right)$ is energy of the bulk WB_5_ and *μ* is a chemical potential of a given element. *S* is the surface area of the slab. For W- and B- terminated surfaces the *n* and *k* could be positive, negative or zero depending on the slab compositions relative to the bulk. Relaxation of simulated slabs was carried out until the total energy difference and the atomic net forces become less than 1 × 10^–5^ eV and 1 × 10^–4^ eV/Å, respectively, to ensure high accuracy and convergence of the calculations.

Computed surface energies were used in Python WulffPack module^91^ for Wulff construction, to obtain the different equilibrium shapes of tungsten boride single crystals. Recent development^92^enable Wulff shape prediction even for low symmetry crystals when surface energies cannot be defined or calculated.

Adsorption energies were calculated by using spin-polarized generalized gradient approximation (GGA) with the PBE parametrization^85^. The Grimme dispersion correction (DFT-D3)^93^ was applied to take into account van der Waals interactions between the surfaces and adsorbed molecules. The cutoff energy of plane waves was set to 500 eV. The partition of the first Brillouin zone into a grid of k-points was carried out within the Monkhorst–Pack scheme^87^ with a resolution of 2π × 0.04 Å^−1^. The convergence parameters for energy and forces were set to 1 × 10^–5^ eV and 1 × 10^–4^ eV/Å respectively.

Different atomic arrangements of molecules on the surface were examined, for the chemisorption of atmosphere gases at several adsorption sites: above the B atoms, above the B-B bond, and above the W atom. The adsorbate molecules (CO, CO_2_, H_2_, N_2_, O_2_, NO, NO_2_, H_2_O, NH_3_, SO_2_) were placed at about ~ 1.5 Å from these surface sites. Adsorbate on one side of the slab and half of the surface layers were fully relaxed, while the rest of the layers beneath remained fixed. For each molecule, its adsorption energy was calculated as:

2 $${E}_{ads}={E}_{calc}-{E}_{slab}-{E}_{m},$$

where ${E}_{calc}$ is the energy of the slab with adsorbed molecule, ${E}_{slab}$ is the energy of the pure substrate, ${E}_{m}$ is the energy of the gas molecule, where *m* denotes the type of the molecule.

The energy barriers for CO and O_2_ adsorption, and energy barriers for CO elementary steps in its oxidation, were obtained by the climbing-image nudged elastic band method, CI-NEB^94^.

### Supplementary Information

Supplementary Information.

## Data Availability

The datasets generated and analysed during the current study are available in the Gitlab repository, https://github.com/AlexanderKvashnin/WB5_surfaces.git.
