# Supplementary material for: Theoretical study of adsorption properties and CO oxidation reaction on surfaces of higher tungsten boride
Source: Sci Rep. 2024 Jun 4;14:12788. doi: 10.1038/s41598-024-63676-7 (PMC11150424; doi:10.1038/s41598-024-63676-7)
Supplement: Supplementary file 1 — Supplementary Information. [file 41598_2024_63676_MOESM1_ESM.docx]

Supporting Information

Adsorption Properties and CO oxidation on Higher Tungsten Boride from First Principles

*Aleksandra D. Radina^1^, Viktor S. Baidyshev^1^, Ilya V. Chepkasov^1^, Nikita A. Matsokin^1^, Tariq Altalhi^2^, Boris I. Yakobson^3,2^ and Alexander G. Kvashnin^1^*

*^1^ Skolkovo Institute of Science and Technology, 121205, Bolshoi Blv. 30, Building 1, Moscow, Russia*

*^2^ Chemistry Department, Taif University, Al Hawiyah, Taif, 26571, Saudi Arabia*

*^3^ Department of Materials Science and NanoEngineering, Rice University, Houston, Texas, 77005, United States*

[1. Stable surfaces and morphology of WB_5_ 2](#_Toc162363287)

[2. Energy barriers of adsorption of O_2_ and CO on surfaces of WB_5_ 5](#_Toc162363288)

[3. Adsorption sites and energies 6](#_Toc162363289)

[References 17](#_Toc162363290)

# Stable surfaces and morphology of WB_5_

Boron-terminated or boron-rich (001) surface can be described with complex armchair-like structure along [010] direction, while W-terminated (001) surface is relatively flat without large differences in height on the surface (see Figure S1a). (010) surface has either atomically flat graphene-like layer of boron atoms or tungsten layer with inclusion of boron triangles (see Figure S1b) due to the structural peculiarity, i.e. boron triangles in bulk WB_5_ occupied one third of 2*b* Wyckoff positions of W in the tungsten (see Ref. ^1^ for details).

Table S1. Structural information about considered surfaces of WB_5_. Miller indices, type of surface termination (mixed termination means that there are both W and B atoms present on the surface), number of tungsten and boron atoms in the slab, lattice parameters of considered slab, calculated surface energies in meV/Å^2^ and J/m^2^.

| Miller indices | Surface termination | N_W_ | N_B_ | Lattice parameters, Å | | Surface energy, meV/Å^2^ | Surface energy, J/m^2^ |
| --- | --- | --- | --- | --- | --- | --- | --- |
| 001 | W | 7 | 34 | 5.2 | 12.737 | 0.122 | 1.958 |
|  | B | 10 | 60 | 5.196 | 12.748 | 0.051 | 0.811 |
| 010 | W | 12 | 48 | 8.994 | 10.400 | 0.134 | 2.153 |
|  | B | 9 | 57 | 8.994 | 10.400 | 0.070 | 1.127 |
| 100 | W | 12 | 44 | 6.368 | 17.986 | 0.135 | 2.164 |
|  | B | 9 | 59 | 6.368 | 17.986 | 0.095 | 1.526 |
| 101 | W | 18 | 90 | 10.387 | 12.748 | 0.046 | 0.734 |
|  | B | 14 | 90 | 10.387 | 12.748 | 0.084 | 1.349 |
| 110 | W | 16 | 69 | 8.221 | 17.986 | 0.170 | 2.722 |
|  | B | 14 | 90 | 8.221 | 17.986 | 0.100 | 1.606 |
| 111 | mixed | 12 | 60 | 8.221 | 22.039 | 0.187 | 2.991 |
| 130 | mixed | 30 | 150 | 16.849 | 17.986 | 0.203 | 3.250 |
| 201 | mixed | 24 | 120 | 18.722 | 12.737 | 0.101 | 1.610 |

Schematic views of (100) surfaces with boron and tungsten terminations are shown in Figure S1c where the graphene-like boron layers located perpendicular to the surfaces. Here we have also distinguished B-terminated and W-terminated surfaces, where W-terminated (100) surface contains of only tungsten atoms (see Figure S1c) and is potentially interesting for catalysis.

Boron terminated (101) surface is like (001) with only exception related to mutual arrangement of boron triangles (see Figure S1d). In the structure of B-(001) surfaces all the triangles on the surface have similar orientation, while in the structure of B-(101) surface there is an alternation of upward and downward oriented boron triangles. Such local changes in the surface may significantly influence the surface energy, stability, and the equilibrium morphology of WB_5_ single crystal.

The B-(110) surface has a saw-like structure where graphene-like boron layers are directed 45 degrees with respect to the surface (see Figure S1e). W-terminated surface in this case contains of almost flat layers of tungsten atoms. The rest of considered surfaces, namely (111), (130), (201) cannot be directly attributed to W-, or B-termination (see Figure S1f-h) due to their complex surface structure. In these slabs, different complex structures made of boron and tungsten atoms are interspersed rather chaotically.


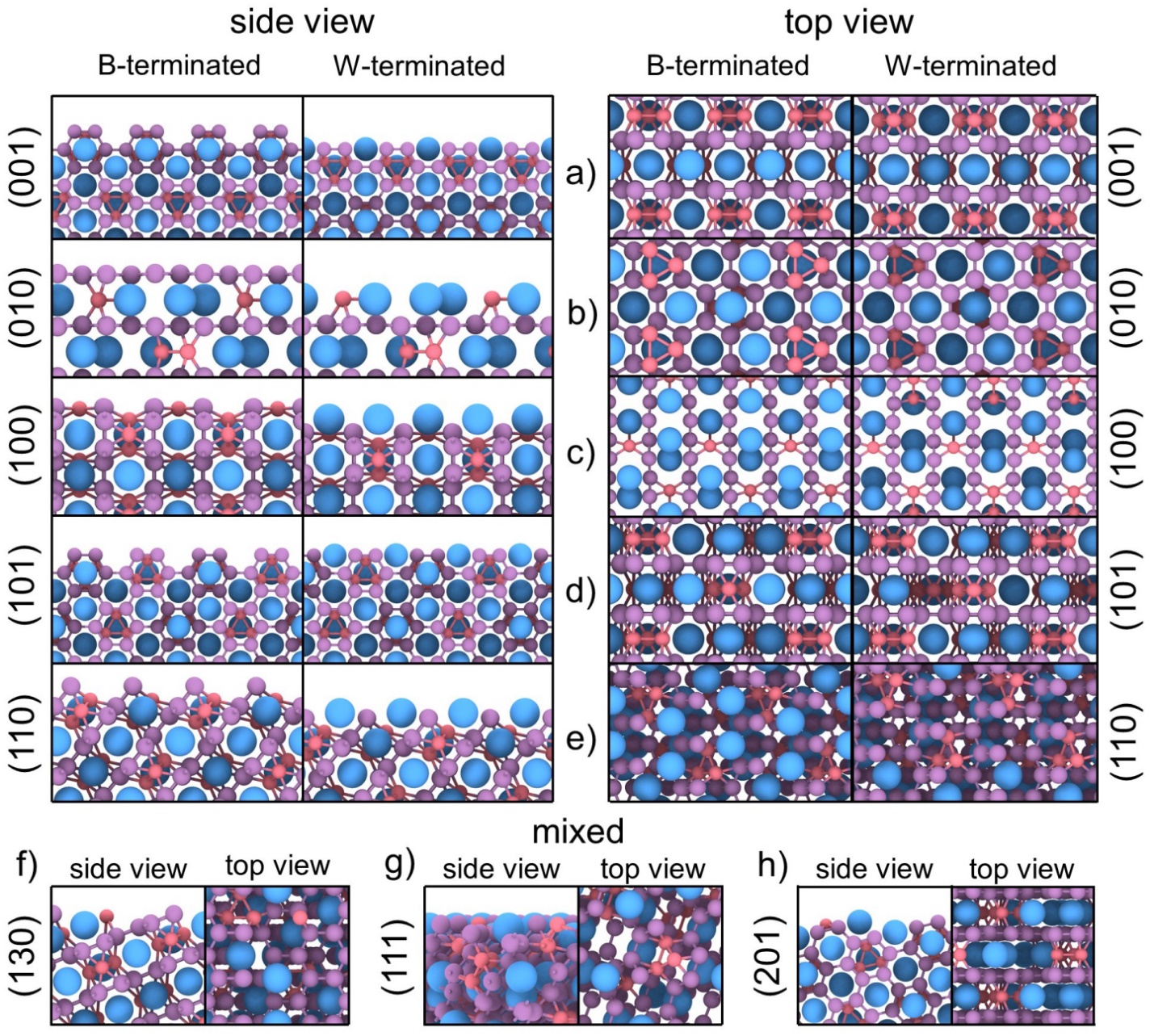


**Figure S1. Schematic side and top views of considered surfaces of WB_5_, namely a) (001), b) (010), c) (100), d) (110), e) (101), f) (111), g) (130), and h) (201). For all surfaces except the mixed ones both terminations with W and B atoms are shown. Tungsten atoms are shown by big blue balls, while boron atoms are shown by small pink and orange balls corresponding to atoms of hexagonal layer and boron triangles respectively.**

For each of considered surfaces we have calculated the surface energy presented in Table S1.

We considered here the surfaces cut from the WB_5_ single crystal of Pmmn space group, predicted to be stable and comprehensively studied earlier^11,20,32^. Slabs of various crystallographic orientations constructed, with thickness 10-16 Å. The (001), (010), (100), (110), (101), (111), (130), and (201) surfaces were constructed for further consideration. For each of (001), (010), (100), (110), (101), two slabs were considered having either B or W termination. Structural information about considered surfaces and descriptions of each surface are presented in the Table S1 and Figure S1.

This data was used in the Wulff construction, for the thermodynamic equilibrium crystal shape at its minimized surface energy^37^. We note that for low symmetry crystals Eq. (1) fails and, moreover, surface energy γ is fundamentally undefinable; but recent theory shows how to circumvent the energy gauge invariance, and to predict crystal morphology, even with unknowable surface energy^40^. The Wulff construction identifies surfaces composition that would be more exposed and the shape of a WB_5_ single crystal, as shown in Fig. S2.

In order to get more information about the stable surfaces and shape of the considered crystals, the influence of the chemical potential was considered by Eq. (2). It should be noted that the shape with μ(B)=-6.89 eV could be obtained only by synthesis with excess amount of tungsten, however these conditions are impossible because WB_5_ phase could be synthesized only in case of excess amount of boron, otherwise the WB_2_ phase would be obtained. Therefore, the phase with μ(B)= -6.68 eV is the closest to reality and would be used for further investigation.


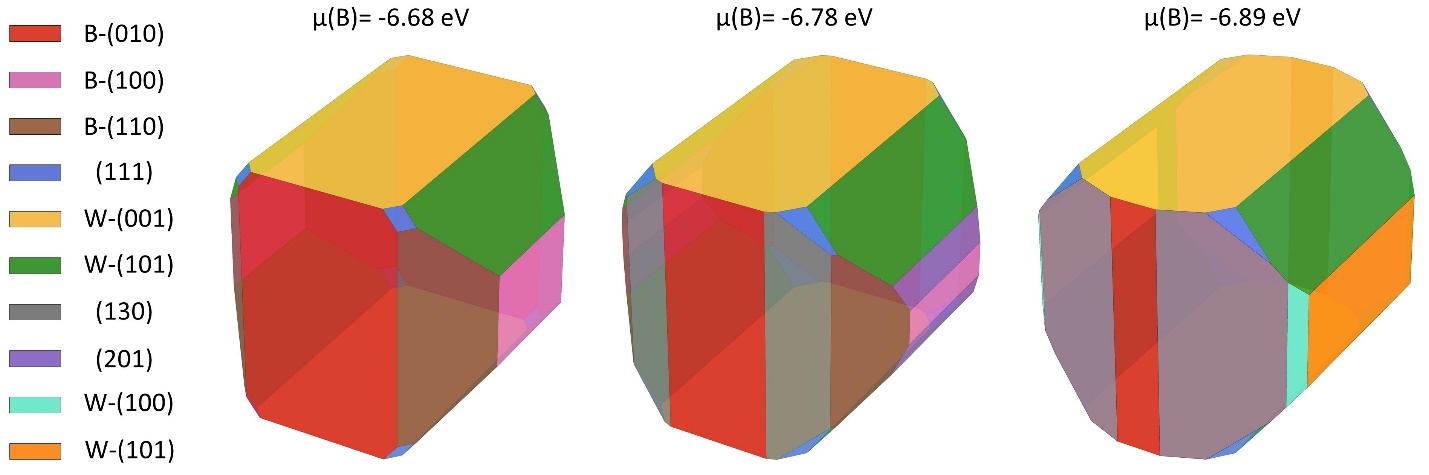


Figure S2. Wulff constructions of WB_5_ single crystal with denoted stable surfaces. Tungsten atoms are shown by big blue balls, while boron atoms are small pink balls.

It should be noted that many of the stable surfaces have similar percent from whole surface area of a crystal: B-(010) – 27.74%, W-(101) – 22.01%, B-(110) – 21.02%, W-(001) – 20.81%, B-(100) – 5.59%, and mixed (111) – 2.83%. In the present study, we considered two surfaces with the highest area. No stable reconstructions were found for these surfaces, which is rational given the honeycomb-like boron structures of the B-(010) surface are very stable and the W-(101) surface is flat enough to be stable. The W-(101) surface contains a high concentration of tungsten atoms and may be more active. However, it is important to consider the surface with high boron content to determine the influence of the boron sublattice on the adsorption and reaction processes. Additionally, the boron content on the surface has a positive effect on the activity in HER and CO_2_ reduction reactions. Therefore, the B-(010) surface could be a potential catalyst for the CO oxidation process.

# Energy barriers of adsorption of O_2_ and CO on surfaces of WB_5_

**
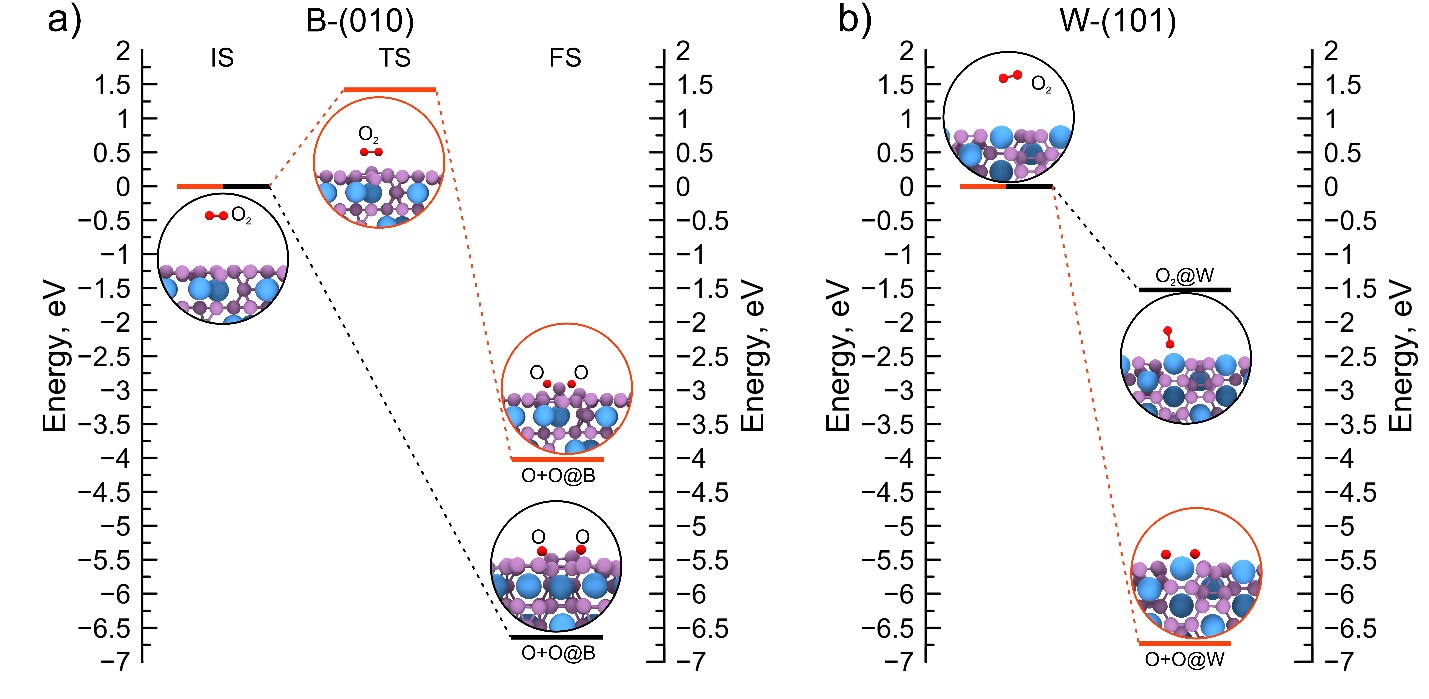
**

Figure S3. Energy barriers for adsorption of O_2_ molecule on the a) B-(010) and b) W-(101) surfaces. Two possible pathways for each surface are shown by black and red colors

**
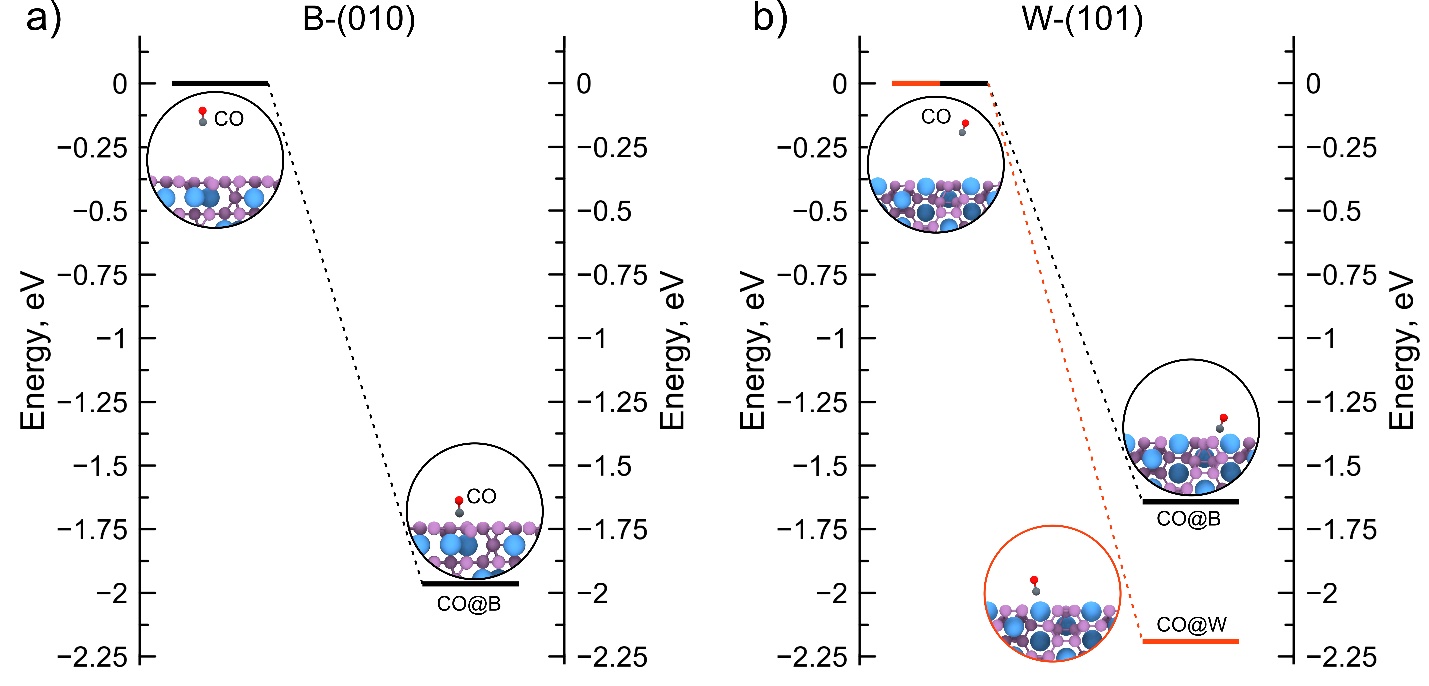
**

Figure S4. Energy barriers for adsorption of CO molecule on the a) B-(010) and b) W-(101) surfaces. The only barrierless option is shown for B-(010). while two possible pathways are shown by black and red colors for W-(101) surface.

# Adsorption sites and energies


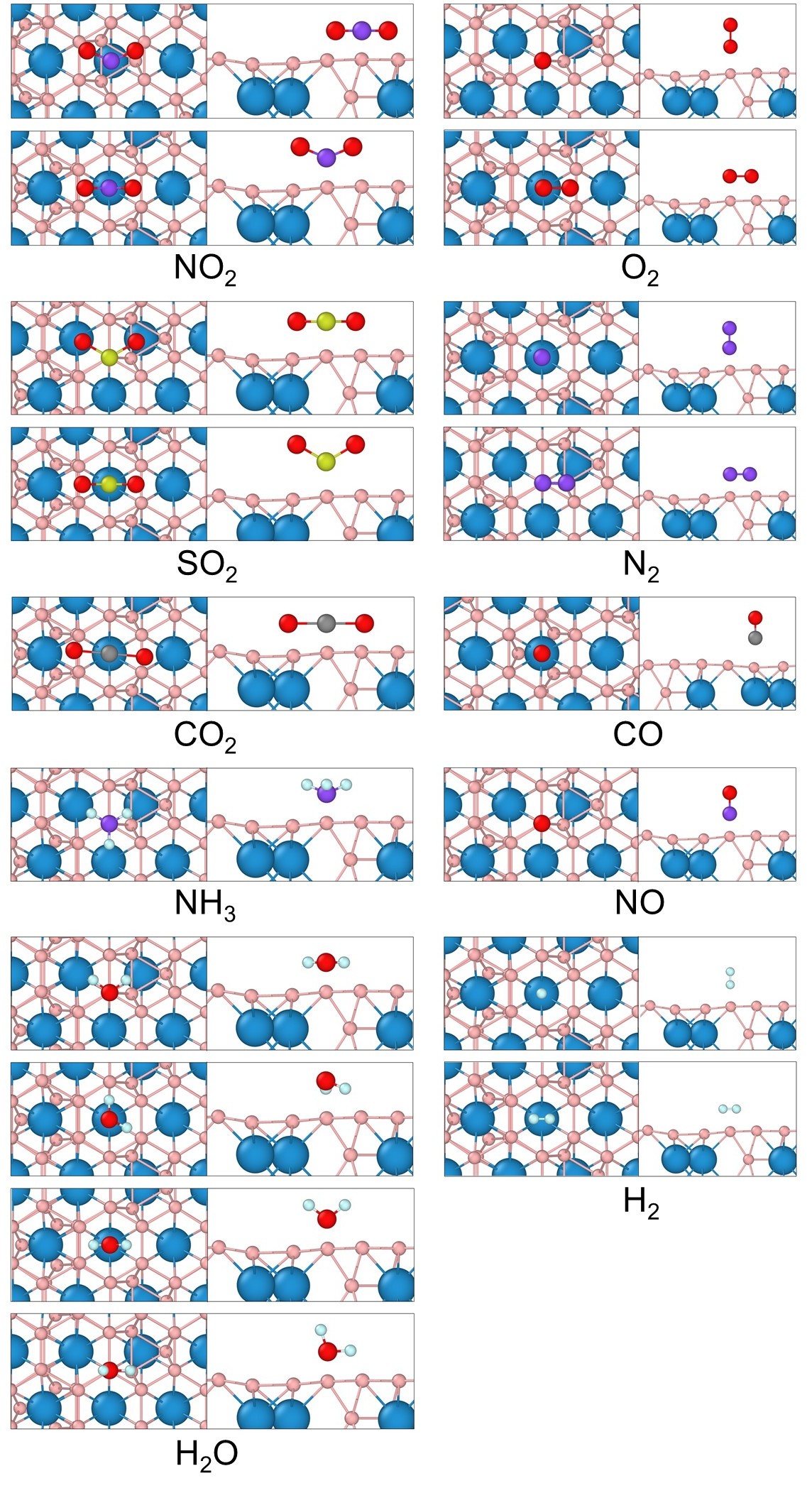


Figure S5. Possible adsorption configurations of the different molecules.

Table S2. Adsorption energies and sites of considered molecules on the surface B-(010) of WB_5-x_. Atomic structure of each configuration can be found at GitHub (<https://github.com/AlexanderKvashnin/WB5_surfaces.git>) using the filename

| **Molecule** | **№ sample** | **Filename** | | **E_ads_** |
| --- | --- | --- | --- | --- |
|  |  | **Input** | **Output** |  |
| **CO** | 1 | CO_1_010_start.vasp | CO_1_010_end.vasp | -1.917 |
|  | 2 | CO_2_010_start.vasp | CO_2_010_end.vasp | -1.968 |
|  | 3 | CO_3_010_start.vasp | CO_3_010_end.vasp | -1.970 |
|  | 4 | CO_4_010_start.vasp | CO_4_010_end.vasp | -2.255 |
|  | 5 | CO_5_010_start.vasp | CO_5_010_end.vasp | -2.066 |
|  | 6 | CO_6_010_start.vasp | CO_6_010_end.vasp | -2.060 |
|  | 7 | CO_7_010_start.vasp | CO_7_010_end.vasp | -1.829 |
| **CO_2_** | 1 | CO2_1_010_start.vasp | CO2_1_010_end.vasp | -0.243 |
|  | 2 | CO2_2_010_start.vasp | CO2_2_010_end.vasp | 0.739 |
|  | 3 | CO2_3_010_start.vasp | CO2_3_010_end.vasp | 0.734 |
|  | 4 | CO2_4_010_start.vasp | CO2_4_010_end.vasp | -0.201 |
|  | 5 | CO2_5_010_start.vasp | CO2_5_010_end.vasp | -1.797 |
|  | 6 | CO2_6_010_start.vasp | CO2_6_010_end.vasp | -0.809 |
|  | 7 | CO2_7_010_start.vasp | CO2_7_010_end.vasp | -1.931 |
|  | 8 | CO2_8_010_start.vasp | CO2_8_010_end.vasp | -0.872 |
|  | 9 | CO2_9_010_start.vasp | CO2_9_010_end.vasp | -1.923 |
|  | 10 | CO2_10_010_start.vasp | CO2_10_010_end.vasp | 2.780 |
|  | 11 | CO2_11_010_start.vasp | CO2_11_010_end.vasp | -0.796 |
|  | 12 | CO2_12_010_start.vasp | CO2_12_010_end.vasp | -2.037 |
|  | 13 | CO2_13_010_start.vasp | CO2_13_010_end.vasp | -0.308 |
|  | 14 | CO2_14_010_start.vasp | CO2_14_010_end.vasp | 3.978 |
|  | 15 | CO2_15_010_start.vasp | CO2_15_010_end.vasp | -0.799 |
|  | 16 | CO2_16_010_start.vasp | CO2_16_010_end.vasp | -0.536 |
|  | 17 | CO2_17_010_start.vasp | CO2_17_010_end.vasp | 0.707 |
|  | 18 | CO2_18_010_start.vasp | CO2_18_010_end.vasp | -0.105 |
|  | 19 | CO2_19_010_start.vasp | CO2_19_010_end.vasp | -0.750 |
|  | 20 | CO2_20_010_start.vasp | CO2_20_010_end.vasp | -0.583 |
|  | 21 | CO2_21_010_start.vasp | CO2_21_010_end.vasp | -0.564 |
|  | 22 | CO2_22_010_start.vasp | CO2_22_010_end.vasp | -0.489 |
|  | 23 | CO2_23_010_start.vasp | CO2_23_010_end.vasp | -0.377 |
|  | 24 | CO2_24_010_start.vasp | CO2_24_010_end.vasp | -0.539 |
|  | 25 | CO2_25_010_start.vasp | CO2_25_010_end.vasp | 0.794 |
|  | 26 | CO2_26_010_start.vasp | CO2_26_010_end.vasp | -1.760 |
|  | 27 | CO2_27_010_start.vasp | CO2_27_010_end.vasp | -0.540 |
|  | 28 | CO2_28_010_start.vasp | CO2_28_010_end.vasp | -0.548 |
| **H_2_** | 1 | H2_1_010_start.vasp | H2_1_010_end.vasp | -4.928 |
|  | 2 | H2_2_010_start.vasp | H2_2_010_end.vasp | -4.583 |
|  | 3 | H2_3_010_start.vasp | H2_3_010_end.vasp | -4.630 |
|  | 4 | H2_4_010_start.vasp | H2_4_010_end.vasp | -4.942 |
|  | 5 | H2_5_010_start.vasp | H2_5_010_end.vasp | -4.960 |
|  | 6 | H2_6_010_start.vasp | H2_6_010_end.vasp | -4.580 |
|  | 7 | H2_7_010_start.vasp | H2_7_010_end.vasp | -4.921 |
|  | 8 | H2_8_010_start.vasp | H2_8_010_end.vasp | -6.380 |
|  | 9 | H2_9_010_start.vasp | H2_9_010_end.vasp | -7.415 |
|  | 10 | H2_10_010_start.vasp | H2_10_010_end.vasp | -4.586 |
|  | 11 | H2_11_010_start.vasp | H2_11_010_end.vasp | -4.627 |
|  | 12 | H2_12_010_start.vasp | H2_12_010_end.vasp | -7.371 |
|  | 13 | H2_13_010_start.vasp | H2_13_010_end.vasp | -6.298 |
|  | 14 | H2_14_010_start.vasp | H2_14_010_end.vasp | -6.691 |
|  | 15 | H2_15_010_start.vasp | H2_15_010_end.vasp | -4.988 |
|  | 16 | H2_16_010_start.vasp | H2_16_010_end.vasp | -6.313 |
|  | 17 | H2_17_010_start.vasp | H2_17_010_end.vasp | -7.035 |
|  | 18 | H2_18_010_start.vasp | H2_18_010_end.vasp | -6.431 |
| **O_2_** | 1 | O2_1_010_start.vasp | O2_1_010_end.vasp | -6.499 |
|  | 2 | O2_2_010_start.vasp | O2_2_010_end.vasp | -3.995 |
|  | 3 | O2_3_010_start.vasp | O2_3_010_end.vasp | 1.532 |
|  | 4 | O2_4_010_start.vasp | O2_4_010_end.vasp | -2.689 |
|  | 5 | O2_5_010_start.vasp | O2_5_010_end.vasp | -4.709 |
|  | 6 | O2_6_010_start.vasp | O2_6_010_end.vasp | -3.809 |
|  | 7 | O2_7_010_start.vasp | O2_7_010_end.vasp | 0.775 |
|  | 8 | O2_8_010_start.vasp | O2_8_010_end.vasp | -5.697 |
|  | 9 | O2_9_010_start.vasp | O2_9_010_end.vasp | -5.894 |
|  | 10 | O2_10_010_start.vasp | O2_10_010_end.vasp | -4.309 |
|  | 11 | O2_11_010_start.vasp | O2_11_010_end.vasp | -4.309 |
|  | 12 | O2_12_010_start.vasp | O2_12_010_end.vasp | -4.790 |
|  | 13 | O2_13_010_start.vasp | O2_13_010_end.vasp | -4.867 |
|  | 14 | O2_14_010_start.vasp | O2_14_010_end.vasp | -2.277 |
|  | 15 | O2_15_010_start.vasp | O2_15_010_end.vasp | -2.502 |
|  | 16 | O2_16_010_start.vasp | O2_16_010_end.vasp | -5.404 |
|  | 17 | O2_17_010_start.vasp | O2_17_010_end.vasp | -5.000 |
|  | 18 | O2_18_010_start.vasp | O2_18_010_end.vasp | -4.040 |
|  | 19 | O2_19_010_start.vasp | O2_19_010_end.vasp | -4.040 |
|  | 20 | O2_20_010_start.vasp | O2_20_010_end.vasp | -5.132 |
|  | 21 | O2_21_010_start.vasp | O2_21_010_end.vasp | -5.011 |
| **NO** | 1 | NO_1_010_start.vasp | NO__010_end.vasp | -9.875 |
|  | 2 | NO_2_010_start.vasp | NO__010_end.vasp | -8.477 |
|  | 3 | NO_3_010_start.vasp | NO__010_end.vasp | -9.177 |
|  | 4 | NO_4_010_start.vasp | NO__010_end.vasp | -9.750 |
|  | 5 | NO_5_010_start.vasp | NO__010_end.vasp | -9.809 |
|  | 6 | NO_6_010_start.vasp | NO__010_end.vasp | -9.886 |
|  | 7 | NO_7_010_start.vasp | NO__010_end.vasp | -8.581 |
| **NH_3_** | 1 | NH3_1_010_start.vasp | NH3_1_010_end.vasp | -1.327 |
|  | 1.1 | NH3_1-1_010_start.vasp | NH3_1-1_010_end.vasp | -1.532 |
|  | 2 | NH3_2_010_start.vasp | NH3_2_010_end.vasp | -0.542 |
|  | 3 | NH3_3_010_start.vasp | NH3_3_010_end.vasp | -0.493 |
|  | 4 | NH3_4_010_start.vasp | NH3_4_010_end.vasp | -2.088 |
|  | 5 | NH3_5_010_start.vasp | NH3_5_010_end.vasp | -2.090 |
|  | 6 | NH3_6_010_start.vasp | NH3_6_010_end.vasp | -0.358 |
|  | 7 | NH3_7_010_start.vasp | NH3_7_010_end.vasp | -1.856 |
| **N_2_** | 1 | N2_1_010_start.vasp | N2_1_010_end.vasp | 0.123 |
|  | 2 | N2_2_010_start.vasp | N2_2_010_end.vasp | -0.041 |
|  | 3 | N2_3_010_start.vasp | N2_3_010_end.vasp | -0.128 |
|  | 4 | N2_4_010_start.vasp | N2_4_010_end.vasp | -0.838 |
|  | 5 | N2_5_010_start.vasp | N2_5_010_end.vasp | -0.681 |
|  | 6 | N2_6_010_start.vasp | N2_6_010_end.vasp | -0.079 |
|  | 7 | N2_7_010_start.vasp | N2_7_010_end.vasp | -0.482 |
|  | 8 | N2_8_010_start.vasp | N2_8_010_end.vasp | -0.377 |
|  | 8.1 | N2_8-1_010_start.vasp | N2_8-1_010_end.vasp | -0.051 |
|  | 9 | N2_9_010_start.vasp | N2_9_010_end.vasp | -0.818 |
|  | 9.1 | N2_9-1_010_start.vasp | N2_9-1_010_end.vasp | 0.414 |
|  | 10 | N2_10_010_start.vasp | N2_10_010_end.vasp | 0.400 |
|  | 11 | N2_11_010_start.vasp | N2_11_010_end.vasp | -0.039 |
|  | 12 | N2_12_010_start.vasp | N2_12_010_end.vasp | 0.297 |
|  | 13 | N2_13_010_start.vasp | N2_13_010_end.vasp | -0.131 |
|  | 14 | N2_14_010_start.vasp | N2_14_010_end.vasp | -0.770 |
|  | 15 | N2_15_010_start.vasp | N2_15_010_end.vasp | -0.497 |
|  | 16 | N2_16_010_start.vasp | N2_16_010_end.vasp | -0.491 |
|  | 17 | N2_17_010_start.vasp | N2_17_010_end.vasp | -0.500 |
|  | 18 | N2_18_010_start.vasp | N2_18_010_end.vasp | 0.625 |
|  | 19 | N2_19_010_start.vasp | N2_19_010_end.vasp | 0.570 |
|  | 20 | N2_20_010_start.vasp | N2_20_010_end.vasp | 0.411 |
|  | 21 | N2_21_010_start.vasp | N2_21_010_end.vasp | 1.085 |
| **NO_2_** | 1 | NO2_1_010_start.vasp | NO2_1_010_end.vasp | -1.329 |
|  | 2 | NO2_2_010_start.vasp | NO2_2_010_end.vasp | -1.250 |
|  | 3 | NO2_3_010_start.vasp | NO2_3_010_end.vasp | -4.575 |
|  | 4 | NO2_4_010_start.vasp | NO2_4_010_end.vasp | -5.592 |
|  | 5 | NO2_5_010_start.vasp | NO2_5_010_end.vasp | -4.315 |
|  | 6 | NO2_6_010_start.vasp | NO2_6_010_end.vasp | -7.673 |
|  | 7 | NO2_7_010_start.vasp | NO2_7_010_end.vasp | -7.830 |
|  | 8 | NO2_8_010_start.vasp | NO2_8_010_end.vasp | -7.166 |
|  | 9 | NO2_9_010_start.vasp | NO2_9_010_end.vasp | -6.919 |
|  | 10 | NO2_10_010_start.vasp | NO2_10_010_end.vasp | -4.094 |
|  | 11 | NO2_11_010_start.vasp | NO2_11_010_end.vasp | -5.080 |
|  | 12 | NO2_12_010_start.vasp | NO2_12_010_end.vasp | -5.075 |
|  | 13 | NO2_13_010_start.vasp | NO2_13_010_end.vasp | -7.611 |
|  | 14 | NO2_14_010_start.vasp | NO2_14_010_end.vasp | -4.781 |
|  | 15 | NO2_15_010_start.vasp | NO2_15_010_end.vasp | -2.267 |
|  | 16 | NO2_16_010_start.vasp | NO2_16_010_end.vasp | -1.821 |
|  | 17 | NO2_17_010_start.vasp | NO2_17_010_end.vasp | 0.070 |
|  | 18 | NO2_18_010_start.vasp | NO2_18_010_end.vasp | -0.251 |
|  | 19 | NO2_19_010_start.vasp | NO2_19_010_end.vasp | -2.771 |
|  | 20 | NO2_20_010_start.vasp | NO2_20_010_end.vasp | -4.472 |
|  | 21 | NO2_21_010_start.vasp | NO2_21_010_end.vasp | -2.237 |
|  | 22 | NO2_22_010_start.vasp | NO2_22_010_end.vasp | -2.529 |
|  | 23 | NO2_23_010_start.vasp | NO2_23_010_end.vasp | -1.341 |
|  | 24 | NO2_24_010_start.vasp | NO2_24_010_end.vasp | -2.854 |
|  | 25 | NO2_25_010_start.vasp | NO2_25_010_end.vasp | -0.802 |
|  | 26 | NO2_26_010_start.vasp | NO2_26_010_end.vasp | -3.007 |
|  | 27 | NO2_27_010_start.vasp | NO2_27_010_end.vasp | -2.181 |
|  | 28 | NO2_28_010_start.vasp | NO2_28_010_end.vasp | -0.845 |
| **H_2_O** | 1 | H2O_1_010_start.vasp | H2O_1_010_end.vasp | -0.366 |
|  | 1.1 | H2O_1-1_010_start.vasp | H2O_1-1_010_end.vasp | 1.122 |
|  | 2 | H2O_2_010_start.vasp | H2O_2_010_end.vasp | -1.520 |
|  | 2.1 | H2O_2-1_010_start.vasp | H2O_2-1_010_end.vasp | -0.527 |
|  | 3 | H2O_3_010_start.vasp | H2O_3_010_end.vasp | -0.487 |
|  | 4 | H2O_4_010_start.vasp | H2O_4_010_end.vasp | -0.614 |
|  | 5 | H2O_5_010_start.vasp | H2O_5_010_end.vasp | -0.660 |
|  | 6 | H2O_6_010_start.vasp | H2O_6_010_end.vasp | -2.530 |
|  | 7 | H2O_7_010_start.vasp | H2O_7_010_end.vasp | -2.384 |
|  | 8 | H2O_8_010_start.vasp | H2O_8_010_end.vasp | -1.162 |
|  | 9 | H2O_9_010_start.vasp | H2O_9_010_end.vasp | -0.991 |
|  | 10 | H2O_10_010_start.vasp | H2O_10_010_end.vasp | -1.307 |
|  | 11 | H2O_11_010_start.vasp | H2O_11_010_end.vasp | -0.519 |
|  | 12 | H2O_12_010_start.vasp | H2O_12_010_end.vasp | -0.547 |
|  | 13 | H2O_13_010_start.vasp | H2O_13_010_end.vasp | -1.848 |
|  | 14 | H2O_14_010_start.vasp | H2O_14_010_end.vasp | -1.013 |
|  | 15 | H2O_15_010_start.vasp | H2O_15_010_end.vasp | -0.539 |
|  | 16 | H2O_16_010_start.vasp | H2O_16_010_end.vasp | -1.520 |
|  | 17 | H2O_17_010_start.vasp | H2O_17_010_end.vasp | -0.512 |
|  | 18 | H2O_18_010_start.vasp | H2O_18_010_end.vasp | -0.579 |
|  | 19 | H2O_19_010_start.vasp | H2O_19_010_end.vasp | -0.646 |
|  | 20 | H2O_20_010_start.vasp | H2O_20_010_end.vasp | -0.711 |
|  | 21 | H2O_21_010_start.vasp | H2O_21_010_end.vasp | -0.790 |
|  | 22 | H2O_22_010_start.vasp | H2O_22_010_end.vasp | -1.159 |
|  | 23 | H2O_23_010_start.vasp | H2O_23_010_end.vasp | -1.164 |
|  | 24 | H2O_24_010_start.vasp | H2O_24_010_end.vasp | -1.306 |
|  | 25 | H2O_25_010_start.vasp | H2O_25_010_end.vasp | -0.521 |
|  | 26 | H2O_26_010_start.vasp | H2O_26_010_end.vasp | -0.542 |
|  | 27 | H2O_27_010_start.vasp | H2O_27_010_end.vasp | -0.525 |
|  | 28 | H2O_28_010_start.vasp | H2O_28_010_end.vasp | -1.282 |
|  | 29 | H2O_29_010_start.vasp | H2O_29_010_end.vasp | -0.581 |
|  | 30 | H2O_30_010_start.vasp | H2O_30_010_end.vasp | -0.594 |
|  | 31 | H2O_31_010_start.vasp | H2O_31_010_end.vasp | -0.607 |
|  | 32 | H2O_32_010_start.vasp | H2O_32_010_end.vasp | -0.201 |
|  | 33 | H2O_33_010_start.vasp | H2O_33_010_end.vasp | -0.716 |
|  | 34 | H2O_34_010_start.vasp | H2O_34_010_end.vasp | -0.342 |
|  | 35 | H2O_35_010_start.vasp | H2O_35_010_end.vasp | -0.691 |
|  | 36 | H2O_36_010_start.vasp | H2O_36_010_end.vasp | -1.118 |
|  | 37 | H2O_37_010_start.vasp | H2O_37_010_end.vasp | -1.148 |
|  | 38 | H2O_38_010_start.vasp | H2O_38_010_end.vasp | -1.306 |
|  | 39 | H2O_39_010_start.vasp | H2O_39_010_end.vasp | -0.682 |
|  | 40 | H2O_40_010_start.vasp | H2O_40_010_end.vasp | -0.079 |
|  | 41 | H2O_41_010_start.vasp | H2O_41_010_end.vasp | -0.512 |
|  | 42 | H2O_42_010_start.vasp | H2O_42_010_end.vasp | -1.063 |
|  | 43 | H2O_43_010_start.vasp | H2O_43_010_end.vasp | -0.515 |
|  | 43.1 | H2O_43-1_010_start.vasp | H2O_43-1_010_end.vasp | -0.528 |
|  | 44 | H2O_44_010_start.vasp | H2O_44_010_end.vasp | -1.041 |
|  | 44.1 | H2O_44-1_010_start.vasp | H2O_44-1_010_end.vasp | -0.463 |
|  | 45 | H2O_45_010_start.vasp | H2O_45_010_end.vasp | -0.576 |
|  | 46 | H2O_46_010_start.vasp | H2O_46_010_end.vasp | -0.264 |
|  | 47 | H2O_47_010_start.vasp | H2O_47_010_end.vasp | -0.696 |
|  | 48 | H2O_48_010_start.vasp | H2O_48_010_end.vasp | -2.226 |
|  | 49 | H2O_49_010_start.vasp | H2O_49_010_end.vasp | -2.484 |
|  | 50 | H2O_50_010_start.vasp | H2O_50_010_end.vasp | -1.087 |
|  | 51 | H2O_51_010_start.vasp | H2O_51_010_end.vasp | -1.165 |
|  | 52 | H2O_52_010_start.vasp | H2O_52_010_end.vasp | -1.304 |
|  | 52.1 | H2O_52-1_010_start.vasp | H2O_52-1_010_end.vasp | -1.296 |
|  | 53 | H2O_53_010_start.vasp | H2O_53_010_end.vasp | -0.537 |
|  | 54 | H2O_54_010_start.vasp | H2O_54_010_end.vasp | -0.212 |
|  | 55 | H2O_55_010_start.vasp | H2O_55_010_end.vasp | -1.276 |
|  | 56 | H2O_56_010_start.vasp | H2O_56_010_end.vasp | -1.281 |
|  | 56.1 | H2O_56-1_010_start.vasp | H2O_56-1_010_end.vasp | -0.530 |
| **SO_2_** | 1 | SO2_1_010_start.vasp | SO2_1_010_end.vasp | 1.809 |
|  | 1.1 | SO2_2_010_start.vasp | SO2_2_010_end.vasp | 0.545 |
|  | 2 | SO2_3_010_start.vasp | SO2_3_010_end.vasp | 1.362 |
|  | 2.1 | SO2_4_010_start.vasp | SO2_4_010_end.vasp | 1.595 |
|  | 3 | SO2_5_010_start.vasp | SO2_5_010_end.vasp | 2.234 |
|  | 4 | SO2_6_010_start.vasp | SO2_6_010_end.vasp | -0.237 |
|  | 5 | SO2_7_010_start.vasp | SO2_7_010_end.vasp | 0.047 |
|  | 6 | SO2_8_010_start.vasp | SO2_8_010_end.vasp | -2.218 |
|  | 7 | SO2_9_010_start.vasp | SO2_9_010_end.vasp | 1.303 |
|  | 8 | SO2_10_010_start.vasp | SO2_10_010_end.vasp | -0.732 |
|  | 9 | SO2_11_010_start.vasp | SO2_11_010_end.vasp | 1.458 |
|  | 10 | SO2_12_010_start.vasp | SO2_12_010_end.vasp | -0.067 |
|  | 11 | SO2_13_010_start.vasp | SO2_13_010_end.vasp | -1.103 |
|  | 12 | SO2_14_010_start.vasp | SO2_14_010_end.vasp | -2.009 |
|  | 13 | SO2_15_010_start.vasp | SO2_15_010_end.vasp | 2.207 |
|  | 14 | SO2_16_010_start.vasp | SO2_16_010_end.vasp | 1.536 |
|  | 15 | SO2_17_010_start.vasp | SO2_17_010_end.vasp | 2.010 |
|  | 16 | SO2_18_010_start.vasp | SO2_18_010_end.vasp | 1.722 |
|  | 17 | SO2_19_010_start.vasp | SO2_19_010_end.vasp | 2.465 |
|  | 18 | SO2_20_010_start.vasp | SO2_20_010_end.vasp | 2.379 |
|  | 19 | SO2_21_010_start.vasp | SO2_21_010_end.vasp | 2.303 |
|  | 20 | SO2_22_010_start.vasp | SO2_22_010_end.vasp | 1.529 |
|  | 21 | SO2_23_010_start.vasp | SO2_23_010_end.vasp | 1.906 |
|  | 22 | SO2_24_010_start.vasp | SO2_24_010_end.vasp | 1.943 |
|  | 23 | SO2_25_010_start.vasp | SO2_25_010_end.vasp | 1.715 |
|  | 24 | SO2_26_010_start.vasp | SO2_26_010_end.vasp | 1.334 |
|  | 25 | SO2_27_010_start.vasp | SO2_27_010_end.vasp | 2.453 |
|  | 26 | SO2_28_010_start.vasp | SO2_28_010_end.vasp | 2.374 |
|  | 27 | SO2_29_010_start.vasp | SO2_29_010_end.vasp | 1.935 |
|  | 28 | SO2_30_010_start.vasp | SO2_30_010_end.vasp | 2.029 |

Table S3. Adsorption energies and sites of considered molecules on the surface W-(101) of WB_5-x_. Atomic structure of each configuration can be found at GitHub (<https://github.com/AlexanderKvashnin/WB5_surfaces.git>) using the filename

| **Molecule** | **№ sample** | **Filename** | | **E_ads_** |
| --- | --- | --- | --- | --- |
|  |  | **Input** | **Output** |  |
| **CO** | 1 | CO_1_101_start.vasp | CO_1_101_end.vasp | -2.683 |
|  | 2 | CO_2_101_start.vasp | CO_2_101_end.vasp | -1.415 |
|  | 3 | CO_3_101_start.vasp | CO_3_101_end.vasp | -2.680 |
|  | 4 | CO_4_101_start.vasp | CO_4_101_end.vasp | -2.686 |
|  | 5 | CO_5_101_start.vasp | CO_5_101_end.vasp | -0.804 |
|  | 6 | CO_6_101_start.vasp | CO_6_101_end.vasp | -1.151 |
|  | 7 | CO_7_101_start.vasp | CO_7_101_end.vasp | -1.245 |
|  | 8 | CO_8_101_start.vasp | CO_8_101_end.vasp | -1.417 |
|  | 9 | CO_9_101_start.vasp | CO_9_101_end.vasp | -1.146 |
|  | 10 | CO_10_101_start.vasp | CO_10_101_end.vasp | -1.339 |
| **NH_3_** | 1 | NH3_1_101_start.vasp | NH3_1_101_end.vasp | -6.780 |
|  | 2 | NH3_2_101_start.vasp | NH3_2_101_end.vasp | -5.063 |
|  | 3 | NH3_3_101_start.vasp | NH3_3_101_end.vasp | -5.055 |
|  | 4 | NH3_4_101_start.vasp | NH3_4_101_end.vasp | -6.837 |
|  | 5 | NH3_5_101_start.vasp | NH3_5_101_end.vasp | -5.904 |
|  | 6 | NH3_6_101_start.vasp | NH3_6_101_end.vasp | -6.641 |
|  | 7 | NH3_7_101_start.vasp | NH3_7_101_end.vasp | -6.395 |
|  | 8 | NH3_8_101_start.vasp | NH3_8_101_end.vasp | -6.643 |
|  | 9 | NH3_9_101_start.vasp | NH3_9_101_end.vasp | -5.087 |
|  | 10 | NH3_10_101_start.vasp | NH3_10_101_end.vasp | -6.836 |
| **N_2_** | 1 | N2_1_101_start.vasp | N2_1_101_end.vasp | -1.511 |
|  | 2 | N2_2_101_start.vasp | N2_2_101_end.vasp | -0.078 |
|  | 3 | N2_3_101_start.vasp | N2_3_101_end.vasp | -0.067 |
|  | 4 | N2_4_101_start.vasp | N2_4_101_end.vasp | -1.537 |
|  | 5 | N2_5_101_start.vasp | N2_5_101_end.vasp | 0.217 |
|  | 6 | N2_6_101_start.vasp | N2_6_101_end.vasp | -0.375 |
|  | 7 | N2_7_101_start.vasp | N2_7_101_end.vasp | -0.855 |
|  | 8 | N2_8_101_start.vasp | N2_8_101_end.vasp | -0.078 |
|  | 9 | N2_9_101_start.vasp | N2_9_101_end.vasp | -0.093 |
|  | 10 | N2_10_101_start.vasp | N2_10_101_end.vasp | -0.066 |
|  | 11 | N2_11_101_start.vasp | N2_11_101_end.vasp | -0.514 |
|  | 12 | N2_12_101_start.vasp | N2_12_101_end.vasp | -0.086 |
|  | 13 | N2_13_101_start.vasp | N2_13_101_end.vasp | -0.089 |
|  | 14 | N2_14_101_start.vasp | N2_14_101_end.vasp | -1.529 |
|  | 15 | N2_15_101_start.vasp | N2_15_101_end.vasp | -1.508 |
|  | 16 | N2_16_101_start.vasp | N2_16_101_end.vasp | -0.629 |
|  | 17 | N2_17_101_start.vasp | N2_17_101_end.vasp | -0.628 |
|  | 18 | N2_18_101_start.vasp | N2_18_101_end.vasp | -0.127 |
|  | 19 | N2_19_101_start.vasp | N2_19_101_end.vasp | -0.079 |
|  | 20 | N2_20_101_start.vasp | N2_20_101_end.vasp | 0.906 |
|  | 21 | N2_21_101_start.vasp | N2_21_101_end.vasp | -0.087 |
|  | 22 | N2_22_101_start.vasp | N2_22_101_end.vasp | 0.677 |
|  | 23 | N2_23_101_start.vasp | N2_23_101_end.vasp | 2.024 |
|  | 24 | N2_24_101_start.vasp | N2_24_101_end.vasp | -0.112 |
|  | 25 | N2_25_101_start.vasp | N2_25_101_end.vasp | -0.099 |
|  | 26 | N2_26_101_start.vasp | N2_26_101_end.vasp | -0.090 |
|  | 27 | N2_27_101_start.vasp | N2_27_101_end.vasp | -0.095 |
|  | 28 | N2_28_101_start.vasp | N2_28_101_end.vasp | -0.092 |
| **O_2_** | 1 | O2_1_101_start.vasp | O2_1_101_end.vasp | -3.657 |
|  | 2 | O2_2_101_start.vasp | O2_2_101_end.vasp | -4.222 |
|  | 3 | O2_3_101_start.vasp | O2_3_101_end.vasp | -3.660 |
|  | 4 | O2_4_101_start.vasp | O2_4_101_end.vasp | -9.935 |
|  | 5 | O2_5_101_start.vasp | O2_5_101_end.vasp | -9.935 |
|  | 6 | O2_6_101_start.vasp | O2_6_101_end.vasp | -9.935 |
|  | 7 | O2_7_101_start.vasp | O2_7_101_end.vasp | -9.178 |
|  | 8 | O2_8_101_start.vasp | O2_8_101_end.vasp | -4.890 |
|  | 9 | O2_9_101_start.vasp | O2_9_101_end.vasp | -9.200 |
|  | 10 | O2_10_101_start.vasp | O2_10_101_end.vasp | -9.521 |
|  | 11 | O2_11_101_start.vasp | O2_11_101_end.vasp | -9.221 |
|  | 12 | O2_12_101_start.vasp | O2_12_101_end.vasp | -9.116 |
|  | 13 | O2_13_101_start.vasp | O2_13_101_end.vasp | -9.976 |
|  | 14 | O2_14_101_start.vasp | O2_14_101_end.vasp | -9.976 |
|  | 15 | O2_15_101_start.vasp | O2_15_101_end.vasp | -9.267 |
| **CO_2_** | 1 | CO2_1_101_start.vasp | CO2_1_101_end.vasp | 1.200 |
|  | 2 | CO2_2_101_start.vasp | CO2_2_101_end.vasp | -0.159 |
|  | 3 | CO2_3_101_start.vasp | CO2_3_101_end.vasp | -1.331 |
|  | 4 | CO2_4_101_start.vasp | CO2_4_101_end.vasp | -2.531 |
|  | 5 | CO2_5_101_start.vasp | CO2_5_101_end.vasp | -0.197 |
|  | 6 | CO2_6_101_start.vasp | CO2_6_101_end.vasp | -2.148 |
|  | 7 | CO2_7_101_start.vasp | CO2_7_101_end.vasp | -2.082 |
|  | 8 | CO2_8_101_start.vasp | CO2_8_101_end.vasp | -0.586 |
|  | 9 | CO2_9_101_start.vasp | CO2_9_101_end.vasp | -0.160 |
|  | 10 | CO2_10_101_start.vasp | CO2_10_101_end.vasp | -0.195 |
|  | 11 | CO2_11_101_start.vasp | CO2_11_101_end.vasp | -2.331 |
|  | 12 | CO2_12_101_start.vasp | CO2_12_101_end.vasp | -2.897 |
|  | 13 | CO2_13_101_start.vasp | CO2_13_101_end.vasp | -1.983 |
|  | 14 | CO2_14_101_start.vasp | CO2_14_101_end.vasp | -1.983 |
|  | 15 | CO2_15_101_start.vasp | CO2_15_101_end.vasp | -0.487 |
|  | 16 | CO2_16_101_start.vasp | CO2_16_101_end.vasp | -0.073 |
|  | 17 | CO2_17_101_start.vasp | CO2_17_101_end.vasp | -0.148 |
|  | 18 | CO2_18_101_start.vasp | CO2_18_101_end.vasp | -0.171 |
|  | 19 | CO2_19_101_start.vasp | CO2_19_101_end.vasp | -0.114 |
|  | 20 | CO2_20_101_start.vasp | CO2_20_101_end.vasp | -0.073 |
|  | 21 | CO2_21_101_start.vasp | CO2_21_101_end.vasp | -0.181 |
|  | 22 | CO2_22_101_start.vasp | CO2_22_101_end.vasp | -0.102 |
|  | 23 | CO2_23_101_start.vasp | CO2_23_101_end.vasp | -0.123 |
|  | 24 | CO2_24_101_start.vasp | CO2_24_101_end.vasp | -0.172 |
|  | 25 | CO2_25_101_start.vasp | CO2_25_101_end.vasp | -0.157 |
|  | 26 | CO2_26_101_start.vasp | CO2_26_101_end.vasp | -0.189 |
|  | 27 | CO2_27_101_start.vasp | CO2_27_101_end.vasp | -0.193 |
|  | 28 | CO2_28_101_start.vasp | CO2_28_101_end.vasp | -0.121 |
| **NO_2_** | 1 | NO2_1_101_start.vasp | NO2_1_101_end.vasp | -3.942 |
|  | 2 | NO2_2_101_start.vasp | NO2_2_101_end.vasp | -2.940 |
|  | 3 | NO2_3_101_start.vasp | NO2_3_101_end.vasp | -3.023 |
|  | 4 | NO2_4_101_start.vasp | NO2_4_101_end.vasp | -2.705 |
|  | 5 | NO2_5_101_start.vasp | NO2_5_101_end.vasp | -2.738 |
|  | 6 | NO2_6_101_start.vasp | NO2_6_101_end.vasp | -3.403 |
|  | 7 | NO2_7_101_start.vasp | NO2_7_101_end.vasp | -4.866 |
|  | 8 | NO2_8_101_start.vasp | NO2_8_101_end.vasp | -3.586 |
|  | 9 | NO2_9_101_start.vasp | NO2_9_101_end.vasp | -3.023 |
|  | 10 | NO2_10_101_start.vasp | NO2_10_101_end.vasp | -6.940 |
|  | 11 | NO2_11_101_start.vasp | NO2_11_101_end.vasp | -3.326 |
|  | 12 | NO2_12_101_start.vasp | NO2_12_101_end.vasp | -3.283 |
|  | 13 | NO2_13_101_start.vasp | NO2_13_101_end.vasp | -2.706 |
|  | 14 | NO2_14_101_start.vasp | NO2_14_101_end.vasp | -2.614 |
|  | 15 | NO2_15_101_start.vasp | NO2_15_101_end.vasp | -2.707 |
|  | 16 | NO2_16_101_start.vasp | NO2_16_101_end.vasp | -1.657 |
|  | 17 | NO2_17_101_start.vasp | NO2_17_101_end.vasp | -1.627 |
|  | 18 | NO2_18_101_start.vasp | NO2_18_101_end.vasp | -0.490 |
|  | 19 | NO2_19_101_start.vasp | NO2_19_101_end.vasp | 0.021 |
|  | 20 | NO2_20_101_start.vasp | NO2_20_101_end.vasp | -1.719 |
|  | 21 | NO2_21_101_start.vasp | NO2_21_101_end.vasp | -1.720 |
|  | 22 | NO2_22_101_start.vasp | NO2_22_101_end.vasp | -2.235 |
|  | 23 | NO2_23_101_start.vasp | NO2_23_101_end.vasp | -1.442 |
|  | 24 | NO2_24_101_start.vasp | NO2_24_101_end.vasp | -2.537 |
|  | 25 | NO2_25_101_start.vasp | NO2_25_101_end.vasp | -3.073 |
|  | 26 | NO2_26_101_start.vasp | NO2_26_101_end.vasp | -2.941 |
|  | 27 | NO2_27_101_start.vasp | NO2_27_101_end.vasp | -0.369 |
|  | 28 | NO2_28_101_start.vasp | NO2_28_101_end.vasp | -2.614 |
|  | 29 | NO2_29_101_start.vasp | NO2_29_101_end.vasp | -2.670 |
|  | 30 | NO2_30_101_start.vasp | NO2_30_101_end.vasp | -2.707 |
| **H_2_O** | 1 | H2O_1_101_start.vasp | H2O_1_101_end.vasp | -1.292 |
|  | 2 | H2O_2_101_start.vasp | H2O_2_101_end.vasp | -1.136 |
|  | 3 | H2O_3_101_start.vasp | H2O_3_101_end.vasp | -0.216 |
|  | 4 | H2O_4_101_start.vasp | H2O_4_101_end.vasp | -0.168 |
|  | 5 | H2O_5_101_start.vasp | H2O_5_101_end.vasp | -1.303 |
|  | 6 | H2O_6_101_start.vasp | H2O_6_101_end.vasp | -1.351 |
|  | 7 | H2O_7_101_start.vasp | H2O_7_101_end.vasp | -0.178 |
|  | 8 | H2O_8_101_start.vasp | H2O_8_101_end.vasp | -1.180 |
|  | 9 | H2O_9_101_start.vasp | H2O_9_101_end.vasp | -0.216 |
|  | 10 | H2O_10_101_start.vasp | H2O_10_101_end.vasp |  |
|  | 11 | H2O_11_101_start.vasp | H2O_11_101_end.vasp | -0.699 |
|  | 12 | H2O_12_101_start.vasp | H2O_12_101_end.vasp | -1.207 |
|  | 13 | H2O_13_101_start.vasp | H2O_13_101_end.vasp | -0.388 |
|  | 14 | H2O_14_101_start.vasp | H2O_14_101_end.vasp | -0.276 |
|  | 15 | H2O_15_101_start.vasp | H2O_15_101_end.vasp | -0.373 |
|  | 16 | H2O_16_101_start.vasp | H2O_16_101_end.vasp | -1.075 |
|  | 17 | H2O_17_101_start.vasp | H2O_17_101_end.vasp | -1.303 |
|  | 18 | H2O_18_101_start.vasp | H2O_18_101_end.vasp | -0.139 |
|  | 19 | H2O_19_101_start.vasp | H2O_19_101_end.vasp | -0.168 |
|  | 20 | H2O_20_101_start.vasp | H2O_20_101_end.vasp | -1.350 |
|  | 21 | H2O_21_101_start.vasp | H2O_21_101_end.vasp | -1.350 |
|  | 22 | H2O_22_101_start.vasp | H2O_22_101_end.vasp | -0.113 |
|  | 23 | H2O_23_101_start.vasp | H2O_23_101_end.vasp | -0.282 |
|  | 24 | H2O_24_101_start.vasp | H2O_24_101_end.vasp | -0.147 |
|  | 25 | H2O_25_101_start.vasp | H2O_25_101_end.vasp |  |
|  | 26 | H2O_26_101_start.vasp | H2O_26_101_end.vasp | -0.254 |
|  | 27 | H2O_27_101_start.vasp | H2O_27_101_end.vasp | -0.291 |
|  | 28 | H2O_28_101_start.vasp | H2O_28_101_end.vasp | -0.390 |
|  | 29 | H2O_29_101_start.vasp | H2O_29_101_end.vasp | -0.284 |
|  | 30 | H2O_30_101_start.vasp | H2O_30_101_end.vasp | -0.374 |
|  | 31 | H2O_31_101_start.vasp | H2O_31_101_end.vasp | -1.232 |
|  | 32 | H2O_32_101_start.vasp | H2O_32_101_end.vasp | -1.131 |
|  | 33 | H2O_33_101_start.vasp | H2O_33_101_end.vasp | -0.195 |
|  | 34 | H2O_34_101_start.vasp | H2O_34_101_end.vasp | -0.159 |
|  | 35 | H2O_35_101_start.vasp | H2O_35_101_end.vasp | -0.363 |
|  | 36 | H2O_36_101_start.vasp | H2O_36_101_end.vasp | -1.347 |
|  | 37 | H2O_37_101_start.vasp | H2O_37_101_end.vasp | -0.171 |
|  | 38 | H2O_38_101_start.vasp | H2O_38_101_end.vasp | -1.176 |
|  | 39 | H2O_39_101_start.vasp | H2O_39_101_end.vasp | -0.203 |
|  | 40 | H2O_40_101_start.vasp | H2O_40_101_end.vasp | -0.205 |
|  | 41 | H2O_41_101_start.vasp | H2O_41_101_end.vasp | -1.334 |
|  | 42 | H2O_42_101_start.vasp | H2O_42_101_end.vasp | -1.200 |
|  | 43 | H2O_43_101_start.vasp | H2O_43_101_end.vasp | -0.383 |
|  | 44 | H2O_44_101_start.vasp | H2O_44_101_end.vasp | -0.272 |
|  | 45 | H2O_45_101_start.vasp | H2O_45_101_end.vasp | -0.367 |
|  | 46 | H2O_46_101_start.vasp | H2O_46_101_end.vasp |  |
|  | 47 | H2O_47_101_start.vasp | H2O_47_101_end.vasp | -0.182 |
|  | 48 | H2O_48_101_start.vasp | H2O_48_101_end.vasp | -1.149 |
|  | 49 | H2O_49_101_start.vasp | H2O_49_101_end.vasp | -1.153 |
|  | 50 | H2O_50_101_start.vasp | H2O_50_101_end.vasp | -0.155 |
|  | 51 | H2O_51_101_start.vasp | H2O_51_101_end.vasp | -0.210 |
|  | 52 | H2O_52_101_start.vasp | H2O_52_101_end.vasp | -0.174 |
|  | 53 | H2O_53_101_start.vasp | H2O_53_101_end.vasp | -0.197 |
|  | 54 | H2O_54_101_start.vasp | H2O_54_101_end.vasp | -3.324 |
|  | 55 | H2O_55_101_start.vasp | H2O_55_101_end.vasp | -0.251 |
|  | 56 | H2O_56_101_start.vasp | H2O_56_101_end.vasp | -0.185 |
|  | 57 | H2O_57_101_start.vasp | H2O_57_101_end.vasp | -0.161 |
|  | 58 | H2O_58_101_start.vasp | H2O_58_101_end.vasp | -0.298 |
| **H_2_** | 1 | H2_1_101_start.vasp | H2_1_101_end.vasp | -1.281 |
|  | 2 | H2_2_101_start.vasp | H2_2_101_end.vasp | -0.060 |
|  | 3 | H2_3_101_start.vasp | H2_3_101_end.vasp | -0.066 |
|  | 4 | H2_4_101_start.vasp | H2_4_101_end.vasp | -1.297 |
|  | 5 | H2_5_101_start.vasp | H2_5_101_end.vasp | -0.063 |
|  | 6 | H2_6_101_start.vasp | H2_6_101_end.vasp | -2.119 |
|  | 7 | H2_7_101_start.vasp | H2_7_101_end.vasp | -2.691 |
|  | 8 | H2_8_101_start.vasp | H2_8_101_end.vasp | -0.061 |
|  | 9 | H2_9_101_start.vasp | H2_9_101_end.vasp | -0.043 |
|  | 10 | H2_10_101_start.vasp | H2_10_101_end.vasp | -0.056 |
|  | 11 | H2_10_101_start.vasp | H2_10_101_end.vasp | -1.281 |
|  | 12 | H2_10_101_start.vasp | H2_10_101_end.vasp | -0.065 |
|  | 13 | H2_10_101_start.vasp | H2_10_101_end.vasp | -0.065 |
|  | 14 | H2_10_101_start.vasp | H2_10_101_end.vasp | -1.297 |
|  | 15 | H2_10_101_start.vasp | H2_10_101_end.vasp | -0.061 |
|  | 16 | H2_10_101_start.vasp | H2_10_101_end.vasp | -0.056 |
|  | 17 | H2_10_101_start.vasp | H2_10_101_end.vasp | -0.050 |
|  | 18 | H2_10_101_start.vasp | H2_10_101_end.vasp | -0.072 |
|  | 19 | H2_10_101_start.vasp | H2_10_101_end.vasp | -0.053 |
|  | 20 | H2_10_101_start.vasp | H2_10_101_end.vasp | -0.065 |
| **NO** | 1 | NO_1_101_start.vasp | NO_1_101_end.vasp | -3.211 |
|  | 2 | NO_2_101_start.vasp | NO_2_101_end.vasp | -0.648 |
|  | 3 | NO_3_101_start.vasp | NO_3_101_end.vasp | -0.894 |
|  | 4 | NO_4_101_start.vasp | NO_4_101_end.vasp | -3.217 |
|  | 5 | NO_5_101_start.vasp | NO_5_101_end.vasp | -0.385 |
|  | 6 | NO_6_101_start.vasp | NO_6_101_end.vasp | -1.739 |
|  | 7 | NO_7_101_start.vasp | NO_7_101_end.vasp | -1.673 |
|  | 8 | NO_8_101_start.vasp | NO_8_101_end.vasp | -2.144 |
|  | 9 | NO_9_101_start.vasp | NO_9_101_end.vasp | -1.577 |
|  | 10 | NO_10_101_start.vasp | NO_10_101_end.vasp | -0.745 |
| **SO_2_** | 1 | SO2_1_101_start.vasp | SO2_1_101_end.vasp | -3.277 |
|  | 2 | SO2_2_101_start.vasp | SO2_2_101_end.vasp | -2.066 |
|  | 3 | SO2_3_101_start.vasp | SO2_3_101_end.vasp | -4.347 |
|  | 4 | SO2_4_101_start.vasp | SO2_4_101_end.vasp | -6.185 |
|  | 5 | SO2_5_101_start.vasp | SO2_5_101_end.vasp | -2.591 |
|  | 6 | SO2_6_101_start.vasp | SO2_6_101_end.vasp | -2.048 |
|  | 7 | SO2_7_101_start.vasp | SO2_7_101_end.vasp | -3.792 |
|  | 8 | SO2_8_101_start.vasp | SO2_8_101_end.vasp | -2.083 |
|  | 9 | SO2_9_101_start.vasp | SO2_9_101_end.vasp | -2.581 |
|  | 10 | SO2_10_101_start.vasp | SO2_10_101_end.vasp | -1.894 |
|  | 11 | SO2_11_101_start.vasp | SO2_11_101_end.vasp | -2.088 |
|  | 12 | SO2_12_101_start.vasp | SO2_12_101_end.vasp | -2.916 |
|  | 13 | SO2_13_101_start.vasp | SO2_13_101_end.vasp | -5.056 |
|  | 14 | SO2_14_101_start.vasp | SO2_14_101_end.vasp | -6.013 |
|  | 15 | SO2_15_101_start.vasp | SO2_15_101_end.vasp | -4.964 |
|  | 16 | SO2_16_101_start.vasp | SO2_16_101_end.vasp | -1.723 |
|  | 17 | SO2_17_101_start.vasp | SO2_17_101_end.vasp | -1.664 |
|  | 18 | SO2_18_101_start.vasp | SO2_18_101_end.vasp | -1.409 |
|  | 19 | SO2_19_101_start.vasp | SO2_19_101_end.vasp | -0.284 |
|  | 20 | SO2_20_101_start.vasp | SO2_20_101_end.vasp | -0.234 |
|  | 21 | SO2_21_101_start.vasp | SO2_21_101_end.vasp | -1.713 |
|  | 22 | SO2_22_101_start.vasp | SO2_22_101_end.vasp | -0.209 |
|  | 23 | SO2_23_101_start.vasp | SO2_23_101_end.vasp | -0.181 |
|  | 24 | SO2_24_101_start.vasp | SO2_24_101_end.vasp | -1.937 |
|  | 25 | SO2_25_101_start.vasp | SO2_25_101_end.vasp | -0.542 |
|  | 26 | SO2_26_101_start.vasp | SO2_26_101_end.vasp | -0.625 |
|  | 27 | SO2_27_101_start.vasp | SO2_27_101_end.vasp | -0.374 |
|  | 28 | SO2_28_101_start.vasp | SO2_28_101_end.vasp | -0.338 |
|  | 29 | SO2_29_101_start.vasp | SO2_29_101_end.vasp | -0.229 |
|  | 30 | SO2_30_101_start.vasp | SO2_30_101_end.vasp | -0.356 |

# References

(1) Kvashnin, A. G.; Rybkovsky, D. V.; Filonenko, V. P.; Bugakov, V. I.; Zibrov, I. P.; Brazhkin, V. V.; Oganov, A. R.; Osiptsov, A. A.; Zakirov, A. Y. WB_5−x_: Synthesis, Properties, and Crystal Structure—New Insights into the Long-Debated Compound. *Advanced Science* **2020**, 2000775. https://doi.org/10.1002/advs.202000775.
